# A Review of Different Types of Liposomes and Their Advancements as a Form of Gene Therapy Treatment for Breast Cancer

**DOI:** 10.3390/molecules28031498

**Published:** 2023-02-03

**Authors:** Gloria Yi Wei Tseu, Khairul Azfar Kamaruzaman

**Affiliations:** Biotechnology Research Institute, Universiti Malaysia Sabah, Jalan UMS, Kota Kinabalu 88400, Malaysia

**Keywords:** liposome, nanoparticles, gene therapy, breast cancer

## Abstract

Breast cancer incidence and mortality rates have increased exponentially during the last decade, particularly among female patients. Current therapies, including surgery and chemotherapy, have significant negative physical and mental impacts on patients. As a safer alternative, gene therapy utilising a therapeutic gene with the potential to treat various ailments is being considered. Delivery of the gene generally utilises viral vectors. However, immunological reactions and even mortality have been recorded as side effects. As a result, non-viral vectors, such as liposomes, a system composed of lipid bilayers formed into nanoparticles, are being studied. Liposomes have demonstrated tremendous potential due to their limitless ability to combine many functions into a system with desirable characteristics and functionality. This article discusses cationic, anionic, and neutral liposomes with their stability, cytotoxicity, transfection ability, cellular uptake, and limitation as a gene carrier suitable for gene therapy specifically for cancer. Due to the more practical approach of employing electrostatic contact with the negatively charged nucleic acid and the cell membrane for absorption purposes, cationic liposomes appear to be more suited for formulation for gene delivery and therapy for breast cancer treatment. As the other alternatives have numerous complicated additional modifications, attachments need to be made to achieve a functional gene therapy system for breast cancer treatment, which were also discussed in this review. This review aimed to increase understanding and build a viable breast cancer gene therapy treatment strategy.

## 1. Introduction

**Figure 1:**
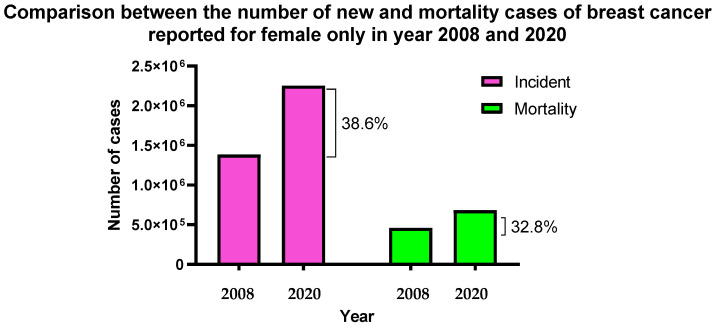
A statistical figure of the number of new breast cancer cases and mortality rate in the female population for the years 2008 and 2020 referring to GLOBOCAN estimated data for years 2008 and 2020 [2,3].

Breast cancer is one of the most common types of cancer in women. In 2020, according to the World Health Organization (WHO), breast cancer ranked the highest number of new cases (11.7%) worldwide for both sexes, with a mortality rate of 6.9% among all cancer types. It reached an incidence rate of 24.5% for females, with the highest mortality rate for all cancers, recording 15% in 2020. Between 2008 and 2020, the number of breast cancer cases in females increased from 1.38 million to 2.254 million, highlighting a 38.6% increase in just over 12 years. The number of deaths within these 12 years has also proportionally increased from 458,400 cases in 2008 to 682,000 cases in 2020, displaying a 32.8% increase [1,2]. These statistical data are summarised in Figure 1. Figure 1 indicates that breast cancer is the most prominent and concerning cancer worldwide, especially for the female population [3]. The consistently increasing numbers of breast cancer cases worldwide have increased the need for effective breast cancer treatment. Bray et al. [4] and Jemal et al. [5] listed several potential factors that have contributed to the increasing number of breast cancer cases worldwide [4,5]. Heredity and genetic factors (family history and breast cancer-causing gene mutation) mean menstruation-related, age of menarche and later age of menopause), reproduction-related (nulliparous, postponement of having firstborn, low rate or reproduction and low rate of breastfeeding), exogenous hormone consumption (oral contraceptive medication and hormone replacement therapy), nutrition (alcohol consumption, high trans-fat content food consumption, and smoking habit), anthropometry (obesity, high weight gain, and body fat distribution) and physical inactivities such as lack of routine exercise or body work out are some of the potential factors that increase the chances of obtaining breast cancer which was summarised in Table 1 [4,5,6]. These factors have been the causes of an increasing number of cases in the past decade. Due to the continuity of today’s lifestyle, these factors remain and are becoming more worrying year by year. 

Breast cancer can be classified into three main histological parts: invasive ductal carcinoma, invasive lobular carcinoma, and mixed ductal-lobular carcinomas [7]. On the surface of cancer cells, receptors are present or overly expressed, such as oestrogen or progesterone receptors (hormonal receptors), HER2 protein receptors, and triple-negative (no molecular receptors listed above). Their presence in cancer cells and not in normal cells play an essential role in a patient’s prognosis and treatment. These receptors were identified and used to study the growth or spread of breast cancer. This enables a better understanding of the potential of discovering cancer drugs or treatments to slow down or even stop cancer growth. The available treatment options that a confirmed breast cancer patient will typically undergo are either surgery, radiation therapy, endocrine therapy, targeted drug therapy, or chemotherapy, which will be used either individually or in a combination of two to three depending on the type and stage of breast cancer [7,8]. Chemotherapy is a go-to treatment in specific situations, such as before or after tumour removal surgery or in advanced stages of the disease; however, they are known for its short- and long-term risks [7,9]. Chemotherapeutic regimens containing anthracyclines (e.g., doxorubicin), taxanes (e.g., paclitaxel), 5-fluorouracil, and cyclophosphamide are high in toxicity as they disrupt DNA replication and mitosis of a patient, which limits their clinical use [7,9]. The most effective available treatment for hormonal receptor breast cancer is targeted oestrogen receptor therapy. However, even with the advancement of these treatments, either solely or combined, their effectiveness is still not guaranteed [10]. Some current clinical treatments, such as cytotoxic chemotherapy and endocrine therapy, show significant promise, but there is still a high possibility of adverse side effects on patients. 

Waks and Winer [7] and Greenlee et al. [11] described that most of the treatments available have several side effects, such as hot flashes, nausea, neutropenia, asthenia, or even sensory neuropathy [7,11]. Patients diagnosed with breast cancer are already physically and emotionally burdened by their illness. Treatments with fewer ambiguities about their adverse effects and efficacy will, without a doubt, relieve patients’ difficulties. Therefore, developing a highly effective and efficient treatment with little to no toxicity or adverse effects is needed. Gene therapy is one such treatment, and it is regarded as having one of the finest potentials for a safe, highly successful, and efficient breast cancer treatment.

Gene therapy is the transfer of genetic material into a patient with the potential to treat various diseases, such as monogenic diseases, infectious diseases, complex neurodegenerative disorders, and cancer, to name a few [12]. Theoretically, gene therapy aims to bring a long-term solution to a patient’s condition, and augmentation gene therapy is one example of a potential method. This can be accomplished by replacing defective genes with copies of mutant or modified genes or by using RNA interference or genome editing techniques to restrict the production of undesirable genes [13]. Anguela and High mentioned that one of the first applied techniques used was direct injection of “naked” nucleic acid which allows transgene expression in the muscle [12]. However, “naked” nucleic acid faces difficulty ensuring successful deliveries to the targeted site and effective response. This is because DNA is large in size, negatively charged, and can easily be degraded by enzymatic nucleases in vivo, thereby preventing efficient delivery. Therefore, a delivery vehicle (vector) must protect and deliver the gene cargo into the target cell or tissue [14]. One of the critical principles of gene therapy’s strategy is to utilise a gene carrier that can protect the genetic material, deliver it, and release it into the targeted site or cell, as genes are pretty fragile, especially when introduced to a foreign biological system [15]. 

One of the first instances of using a vector was performed in the early 1990s. Clinical research on gene therapy was started at the US National Institutes of Health in 1990 to treat a rare inherited immunodeficiency disorder, immunodeficiency–X1 (SCID-X1), which used a complementary DNA with a defective retrovirus-derived vector [16]. However, vector insertional oncogenesis occurred in 5 of 20 patients, resulting in leukaemia, and this treatment came to a halt when it caused the first death. After treatment, a teenager named Jesse Gelsinger died due to systemic inflammatory response syndrome [17]. In a different case, Aiuti et al. [18] found a promising trial for ADA-SCID (adenosine deaminase deficient severe combined immunodeficiency). This disease has a faulty immune function leading to toxic metabolite accumulation that will cause organ damage and, eventually, death [18,19]. Although both cases used viral gene therapy as their treatment, the negative impact of SCID-X1 on the patient was not seen in ADA-SCID patients even after long periods whereby both treatments integrated a similar gene. Since then, more than 2500 clinical studies have been conducted for a broader range of applications for gene therapy as of 2018 [12]. Developments in gene therapy remain slow due to numerous limitations. These challenges include insertional mutagenesis, small cargo capacity, failure to reach inaccessible tumours, immunogenicity cytotoxicity issues, stability problems, enzymatic degradation, and the major unresolved concern of how to effectively and efficiently deliver the therapeutic gene to the targeted site [20]. Despite these complications, there have been numerous advancements in developing techniques and a deep understanding of gene therapy. According to Anguela and High’s [12] findings, six gene therapies, mainly viral therapy and immunotherapy, have already received approval from medical agencies, as summarised in Table 2. Though gene therapy treatments have already been approved, none utilising viral vectors have successfully targeted cancer, especially breast cancer.

There were approved viral gene therapies available in the market, but many cases claimed viral vectors to have severe adverse effects, with one example reporting systemic inflammatory response syndrome, which has led to multiple organ failures in the human host and even caused deaths [15,20]. Therefore, numerous efforts have focused on discovering alternative delivery vehicles or systems. For instance, non-viral nanoparticle delivery systems, including fullerenes and carbon-based, metal, ceramic, semi-conductor, lipid-based, and polymeric nanoparticles, have all shown some potential [22]. Non-viral delivery systems research has been steadily increasing due to its characteristics as a safer and easily customisable system. One such system is liposomes, a lipid-based nanoparticle system, where lipid bilayers encapsulate an exogenous therapeutic cargo to navigate the biological barriers and environment. They are easily assembled, manufactured on a large scale and modified to form a functional non-viral vector. Viral vector manufacturing on a large scale and long-term storage of viral vectors is less stable, resulting in low yields, loss of purity, and short shelf life. Therefore, based on this basis, the liposome is a more preferred vector to a viral vector [23]. 

Bangham et al. [24] at the Babraham Institute, University of Cambridge, first discovered liposomes in the 1960s when they created both single and multiple concentric lipid bilayers encapsulating an aqueous compartment [24]. It was able to entrap lipophilic and hydrophilic agents either on the surface of the lipid membrane or in the aqueous core. The size of these nearly spherical lipid vesicles can range from a few nanometres to several micrometres. Based on Balazs et al. [25], possible shapes formed are spherical micelles, cylindrical micelles, flexible bilayer vesicles, planar bilayers, and inverted micelles [25]. In this review, different types of liposomes (cationic, anionic, and neutral) were discussed as well as the stability, cellular uptake, transfection efficiency, cytotoxicity, and disadvantages of each type of liposome.

Conventional breast cancer therapies are frequently non-selective for tumour cells, resulting in significant toxicity, ineffectiveness, and multiple adverse effects. However, in turn, due to liposomes’ high modification properties, functional liposomes make targeting properties as a form of treatment possible. Due to their low immunogenicity and toxicity compared to viral vectors, liposomes have been the most commonly utilised nonviral vector. Liposomes also show great potential in use as a nanocarrier for targeted treatments for tumour sites, as they can be easily modified by incorporating either a single or a combination of targeting strategies that use a variety of ligands, polymers, or receptors that specifically target breast cancer [26]. Moreover, liposomes can encapsulate not just therapeutic genes but also cytotoxic drugs. The surface modification ability of the liposomes enables higher cargo concentrations to be delivered to the tumour site. 

Furthermore, the presence of the phospholipid bilayer protects the cargo encapsulated from being broken down in the body before reaching tumour tissue and minimises cargo exposure to healthy sensitive tissue [27]. This minimises damage to normal tissues and no longer requires long-term transgene expression, especially in treating breast cancer [28]. Developing a targeted gene therapy treatment system using liposomes as gene carriers for breast cancer has made slow progress in the past years. Understanding each type of liposome’s properties may help formulate a functional liposome for gene therapy for breast cancer. This review addresses recent research that has employed liposomes as a nonviral vector for cancer genes and drug therapy, discussing the main results obtained using in vitro and in vivo studies while paying particular attention to cationic liposomes and ways to overcome their disadvantages over anionic and neutral liposomes.

## 2. Gene Therapy

Each subtype of breast cancer was linked to gene alterations that caused certain cells in the breast to become aberrant. Thus, gene therapy is a viable treatment option for breast cancer subtypes with different genetic abnormalities [29]. Breast cancer susceptibility genes with high-risk variant alleles such as breast cancer susceptibility genes (BRCA1, BRCA2); tumour protein p53 (TP53); phosphatase and tensin homologue (PTEN); serine/Threonine kinase 1 (STK11); and cadherin 1 (CDH1) are some of the genes that will give rise to the relative risk of breast cancer [30]. Gene correction, gene editing, suicide gene therapy, and gene suppression are some strategies chosen for breast cancer gene therapy treatment. 

Transfection refers to the ability of a cell to express the desired nucleic acid or protein that is introduced into the targeted cell. Nucleic acid cannot be introduced into a host cell directly, so a transport vehicle is needed to deliver the gene of interest into a host cell. Genes of interest that can be used for transfection are plasmids, RNA, mRNA, and oligonucleotides. Transfection is impossible with exogenous naked genetic material, such as DNA or RNA, as it will degrade through multiple means as soon as it enters the human system. Opsonisation, rapid clearance by the RES, poor tumour penetration, cellular uptake, and lysosomal degradation are some obstacles that genetic material will have to face before it can successfully transfect a cell [31]. Therefore, a cargo carrier that can encapsulate the genetic materials as a means of protection to ensure the high possibility of the cargo reaching the targeting site successfully is needed. 

The non-viral transfection approach is divided into two methods: physical and chemical transfection [32]. Electroporation, sonoporation, magnetofection, gene microinjection, and laser irradiation are all standard physical transfection methods to introduce genetic material into a host cell using various physical tools [33]. Liposomes, calcium phosphate, dendrimers, polymers, nanoparticles, and non-liposomal lipids are some of the most commercially available chemical transfection reagents. These chemical transfection reagents will help introduce foreign genetic elements into a host cell with minimum chemical resistance. 

There are two types of possible transfections for a non-viral vesicle with the gene of interest encapsulated: stable transfection and transient transfection, depending on the nature of the gene of interest. Genetic material introduced into the cell genome for long-term expression will undergo stable transfection. As for transient transfection, genetic material is not integrated into the host cell genome and is only expressed for a short time [32,33]. Thus, the cargo carrier should have properties that will allow the release of the gene cargo either by degrading or unravelling itself at strategic locations with the highest transfection percentage [15]. The surface of the cargo carrier also plays a vital role in ensuring efficient transfection. Since nucleic acid is negatively charged, it needs a positively charged agent to be its carrier, creating an electrostatic interaction naturally during the encapsulation process [15]. Suppose the cargo carrier has a high positive charge density. In that case, it might attract too strongly to the negatively charged nucleic acid, reduce the transfection efficiency, and even cause aggregation when electrostatic repulsion becomes too low, causing accumulation in the lungs and liver instead of circulating in the circulatory system [31]. 

For targeting function, length and type of aliphatic chains in the lipids, charge density, hydrophobicity, adding polymers or biomolecules, and binding affinity to the protein or receptor of a cell need to be put into consideration. This allows numerous modifications of the surface of the cargo carrier for enhanced desired functions and properties such as biodegradability, charge density, solubility, molecular weight, crystallinity, hydrophobicity, rigidity, and pKa value, all essential towards the effectivity of protection and delivery. Other than that, the size of the cargo carrier complex plays an important role. It will determine the cellular uptake pathway, indicating the surface modification such as surface amines (lysine), sulfhydryls (cysteine), and non-native functionality, such as aldehydes (generated from sugars) and also azides (via metabolic introduction) [34] need to put into consideration for developing the delivery system [35]. All these modifications will be complicated to achieve in a viral transfection system, but it is possible for a liposome-based delivery system due to its robust modification ability. 

## 3. Liposomes 

Liposomes, categorised as a non-viral delivery system, are generally made from phospholipids and consist of a hydrophilic head and two hydrophobic chains. As molecules with both hydrophilic and hydrophobic segments, they have a high possibility of forming membranes when dispersed in an aqueous solution where the polar heads will favour aqueous environments, and their long aliphatic chains will favour interactions with one another. These interactions create van der Waals force and hydrogen bonds in general, forming a hollow-centred lipid bilayer vesicle of different shapes, such as spherical, polyhedral, or tubular, depending on their nature, concentration, temperature, and geometric form [36]. The shape of a liposome is heavily influenced by the type of lipid used in its formulation, as different lipid combinations result in different conformational structures. Liposomes can be customised and designed into different shapes and sizes, either bilayer or monolayer, and even multiple layers for specific functions. For instance, lipid capsules or closed bilayer vesicles are used in cosmetics and drug-delivery industries due to their biologically compatible nature. It can also be designed to be sensitive to the environment, such as temperature or pH, to release its content to a specific site [37]. 

Moosavian and Sahebkar’s work investigating liposomes in cancer cells also found that the liposomes’ elasticity (the liposomes’ ability to squeeze through tiny membrane pores at the surface of a plasma membrane) of liposomes does affect their functionality [38]. The elasticity of a liposome could alter cellular uptake and circulation time, where it can be internalised more and faster than less elastic nanoparticles. Less elastic liposomes interact more readily with cells and enter via the clathrin-mediated pathway, but more elastic liposomes rely on micropinocytosis [38]. Key et al. [39] and Anselmo et al. [40] reported that the elasticity of liposomes affecting the liposome-cell interaction depends very much on the type of tumour cells, and liposomes with lower elasticity interact better with immune cells compared to liposomes with higher elasticity [39,40]. It was proven again by Anselmo et al. [40] that different tumours need to be identified, and the liposomes used need to be tailored accordingly, as elasticity affects biodistribution and tumour accumulation [40]. Due to the variation and the countless means for customisation in their characteristics, such as stability, pharmacokinetic properties, and therapeutic efficacy, researchers have achieved this by modifying the composition, size, charge, and other components of the liposomes [41]. Most formulations comprise zwitterionic, cationic or anionic lipids, PEG, and/or cholesterol, which affect stability, pharmacokinetics, and transport in different ways [42].

Commercially, liposomes are used in cosmetic industries as penetration enhancers, in food industries as solubilisers, as carriers in medical diagnostics, as signal enhancers in analytical biochemistry, and as medicinal vehicles (drugs or genes) [43,44]. Many medication candidates have been encapsulated in liposomes and studied for reduced toxicity and prolonged therapeutic effect duration. This reduces the toxicity of administering patients with just the drug alone. Various academic and industry research groups have established liposomal encapsulation of drugs and genes [45]. Of all the approved liposome-based delivery systems used as treatments against various diseases, Doxil, Lipo-dox, and Myocet were the only approved treatments specifically for breast cancer, as shown in Table 3. Both approved liposome-based treatments use liposomes to encapsulate anthracycline drugs known to have high toxicity if administered directly to patients and cannot exceed a dosage of 450–500 mg/m^2^ per lifetime. Although encapsulating anthracycline with liposome can reduce toxicity and can be administered up to 2200 mg/m^2^ per lifetime to breast cancer patients, patients still encounter side effects such as cardiotoxicity and alopecia [46]. 

More recent advancements have been investigated for optimising liposomal-based delivery systems with more reproducible preparation techniques and a broader application to novel modalities, including nucleic acid therapies, CRISPR/Cas9 therapies, and immunotherapies, to meet the ongoing demand for new treatments in clinics [45]. Liposomes are preferred over viruses in gene-delivery applications, such as gene therapy and gene vaccination. This is because they are non-immunogenic and much easier to assemble than a viral transfection system [50,51]. 

Due to the flaw of the viral transfection system and liposome-aided drug treatment, over the last few decades, researchers have been committed to developing liposome-based gene delivery systems as a better alternative. Thus, there have been a rising number of FDA-approved liposomal-based treatments and an increasing number of clinical trials, including a wide range of applications for a liposome-based gene therapy treatment specifically to treat cancer. Unfortunately, as shown in Table 4, only two clinical trials used liposomes as the carrier for breast cancer gene therapy treatment. 

There are endless potentials and possibilities for developing liposome formulations combining different types of lipids as vesicle entrapping cargos with numerous treatments and strategies with endless possibilities. This review hopes that by looking into and comparing different types of liposomes, cytotoxicity, mechanisms of uptake and transfection efficiency of different diseases to gain a better understanding towards developing a safer and more effective treatment specifically for gene therapy in treating breast cancer. As previously mentioned, there are three main types of liposomes; anionic, cationic, and neutral-charged. They will be discussed in greater detail, including their benefits and drawbacks, features, functioning, and modes of action, to understand better and exploit these many forms of liposomes.

## 4. Cationic Liposomes

Cationic lipids are amphiphilic molecules with three main parts in their molecules, which consist of positive-charged polar heads, a linker and hydrophobic tails [35]. Table 5 shows some of the more common cationic lipids used to formulate cationic liposomes. Each positively charged polar head may contain either a single or multiple positively charged functional groups, which can form complexes with an anionic cargo. Complexes can be formed by putting molecules with positive and negative charges together, forming electrostatic interactions. Hence, the utilisation of dense nucleic acids due to numerous negatively charged phosphate groups tends to spontaneously form condensed cationic lipoplexes when brought together with a positively charged liposome or lipid species [35,51]. Usually, lipoplexes are formulated using excess positively charged lipids, such as in Table 5, to DNA/RNA ratio to create a positively charged complex that will create an interaction between surface proteoglycans and the cationic lipoplex system. This will then help with cell binding, endocytosis, and fusion of anionic endosomal membranes, which triggers the release of genetic materials into the cytoplasm [54]. Other liposomes, compared to cationic liposomes, have lower DNA entrapment efficiency, lower extent of cellular internalisation, and efficiency in protecting DNA in the cytoplasm during nuclear entry [55]. Cationic lipids with cationic headgroups create a natural electrostatic contact between the lipid and the genes, which improves encapsulation efficiency. Moreover, the electrostatic interaction between cationic liposomes and the cell membrane enables cationic liposomes to internalise the cell more efficiently than other liposomes [31,56].

### 4.1. Stability of Cationic Liposomes 

Their size distribution and polydispersity can determine liposomes’ physical stability. Liposome size can be determined using a variety of techniques such as dynamic light scattering, electron microscopy, atomic force microscopy, field flow fractionation, nanoparticle tracking analysis, flow cytometry, fluorescence microscopy, size exclusion chromatography, scanning ion occlusion sensing, centrifugal sedimentation, and differential scanning calorimetry [45]. Cationic liposomes are known to have low serum stability as they tend to be attracted to plasma proteins (serum albumin, complements, immunoglobulins, and apolipoproteins), forming a corona layer on their surface [51]. The serum contains anionic proteins that attract the cationic liposome due to electrostatic interactions when introduced into a host cell [57]. This will tend to dissociate and degrade the genetic materials encapsulated in the cationic liposomes, thereby affecting optimal performance. The positively charged liposomes will also naturally be attracted to other negative charge elements in the host’s body, with the potential to sway the liposome away from the targeted site. 

Furthermore, cationic liposomes also display low activity, especially since cationic liposomes are identified as foreign material within the human host. This will lead to coating by antibodies or phagocytes ingesting instead of reaching the targeted site [31]. However, according to Kapoor M. and Burgess D., it does not affect silencing efficiency, which was attributed to highly efficient endosomal escape ability compared to anionic liposomes [58]. Liposomes formulated with only cationic lipids will form liposomes with a high positive charge and tend to form less-stable micelles with high toxicity. For this reason, formulators are figuring out strategies to reduce the positive charge. This can be accomplished as cationic liposomes can be modified via the addition of ligands such as proteins [59] or polymers [60] to shield part of the charge, adding helper lipids into the formulation to reduce the cationic lipid [55] or formulating single laminal vesicles for smaller particles. These are some of the few strategies to overcome such limitations.

### 4.2. Cytotoxicity of Cationic Liposomes

Cationic liposomes, utilised in nucleic acid delivery, have high toxicity toward macrophages, which reduces immunomodulator secretion and thereby depletes macrophages in the host [61]. Other than that, cationic liposomes tend to activate cellular pathways such as pro-apoptotic and pro-inflammatory cascades, which is a significant drawback to using liposomes in gene delivery and therapy as these will lead to an immunogenic response in the host [62]. However, the toxicity of the head group mechanism is still not fully known, which is where further research is needed to aid in developing liposomes as a non-viral carrier. Simoes et al. [55] carried out some studies, and they found that lipid linkages play an essential role in the cytotoxicity of liposomes, whereby lipids with stable ether linkages (e.g., DOTMA, DMRIE) have higher cytotoxicity than those with labile ester linkages (e.g., DOTAP) [55]. A cationic lipid with ester linkers tends to have lower cytotoxicity than lipids with ether and amide linkers [35]. 

Cationic lipids have the potential to disrupt the anionic cellular membrane, resulting in unfavourable cytotoxicity [45]. The main factors that will affect unfavourable cytotoxicity are the hydrophilic head groups and the morphology of the lipid molecules, such as the length of the hydrophobic chains. Moreover, the type of chemical bonds and the relative position of the hydrocarbon chains can also influence toxicity [63,64]. However, cationic lipids have a significant advantage over anionic and neutral lipids. The positively charged head is easily attracted to the negatively charged cell membranes, which increases the cell incorporation or uptake rate. However, particles that enter the cell by endocytosis will tend to be trapped in the endosome, which will then fuse with the lysosome. Once fused, hydrolytic enzymes will be released, which can break down different types of biomolecules. When highly positively charged particles enter the cell, they will still be trapped in the endosome. Still, because of their high positive charge, it will trigger a “proton sponge effect”, which will lead to the endosome rupture due to the involvement of osmotic pressure, thus releasing the cargo inside [65]. Therefore, the cationic lipid is the most effective compared to anionic and neutral lipids used in liposomes, specifically on cellular uptake [66]. However, the high positive charge is also generally attributed to its downfall, causing significant cell damage. Despite this, cytotoxicity can be successfully reduced with different approaches, modifications, and combinations of different strategies in the formulation stage. Some studies discovered that reducing the charge from the overall formulation (lower zeta potential) reduced the cytotoxicity levels. This involves the addition of neutral or zwitterionic lipids into the cationic liposome formulation to reduce the surface charge of the cationic liposomes, yielding liposomes with lower zeta potential [15,67].

Lipids used in formulating liposomes with a permanent positive charged ammonium head group have a much higher cytotoxic effect when compared to those with a tri-peptide (e.g., tri-arginine) head group [62]. Endogenous amino acids such as arginine [68] have the potential to assist lipid molecules in showing improved transfection efficiency. Their membrane-penetrating ability w be similar to cell-penetrating peptides in cell translocation. Moreover, adding certain polycations, polymers, or peptides into a cationic liposome formulation has increased cell transfection efficiency and reduced cytotoxicity [69,70]. According to Rezaee et al. [70], cationic polymers are often incorporated in formulating gene carriers because they condense, thereby creating smaller-sized particles. Cytotoxicity caused by the addition of polymers into liposome formulations generally causes poor biocompatibility and biodegradability of the system, though there are also strategies to overcome this issue. Another option will be to use peptide-based polycations, which have been claimed to increase their transfection efficiency in cationic liposome formulation. The attachment of peptides also reduced the cytotoxicity of cationic lipids in liposome formulation, but using high molecular weight peptides will increase the immunogenicity of the liposome complex [31]. Attaching polymers such as PEG into cationic liposome formulations is shown to not only be able to shield the positive charge surface density but also increase the stability of the system. 

Cationic polymers can also be attached to neutral liposomes and reduce their particle size and carrier (liposome) to nucleic acid (cargo) encapsulation ratio with low cytotoxicity. Using lower-concentration cationic polymers attached to neutral liposomes to create lower-charged liposomes can compensate for any adverse effect caused by higher-charged cationic liposomes [70]. Many peptides, including cell-penetrating peptides, targeting peptides, nuclear localising peptides, and peptides that stimulate endosomal release, can be joined with a cationic peptide (as a nucleic acid condensing domain) via a cleavable linker to create a multifunctional peptide-based liposome [70]. Rezaee et al. [70] have summarised the use of different polymers and peptides incorporated into liposome formulations. 

Although adding polymers or peptides onto the surface of liposomes can reduce cytotoxicity, it will contribute to the increase in particle size, which is something to be considered. There have been several approaches, such as attaching biocompatible polymers, cleavable polymers that are cleaved to their subunits in the cytosol and endosome, using less cationic polymers (resulting in lower cytotoxicity) that are compensated with more cationic liposome, hydrophobic modifications, and conjugation of some adducts including hydrocarbon chains of varying lengths, and some hydrophobic pharmaceuticals such as glucocorticosteroids, which act as nuclear localisation signals and immunomodulators, (cleavable) PEG, and targeting ligands [70]. The efficiency of different combinations has shown an increase of 2–400 folds compared to liposomes without any attachment. 

Looking into the cytotoxicity of a liposome formulation is crucial as it determines the fundamental requirement for it to work as a carrier that will not affect the viability of cells. Suppose a formulation adversely affects the viability of the cell. In that case, it will contradict its function as a carrier as it will interfere with the treatment of the cargo it carries. Once the cytotoxicity level falls into the acceptable range, a formulation’s cellular uptake and transfection efficiency will need to be investigated to justify further the formulation’s functionality and effectiveness as a carrier for genes or drugs.

### 4.3. Cellular Uptake of Cationic Liposome

Cellular uptake is one of the most critical processes that regulate a molecule’s biological activity upon entering a cell, and interactions between the substance and the plasma membrane of a targeted cell govern it. There are numerous uptake pathways for a molecule to enter a cell. One such pathway involves molecules being taken up by the host cell through endocytosis in membrane invaginations by the engulfment of liposomes, which then leads to budding and pinching off, forming endocytic vesicles [64]. It will then be moved into specialised compartments for intracellular sorting or trafficking depending on the type of cell and molecules (e.g., proteins, lipids) involved in the endocytosis process [71]. Phagocytosis, caveolae or clathrin-independent, caveolae-mediated, clathrin-mediated endocytosis, or macropinocytosis are other possible pathways depending on the size and charge of the liposome complex used. If the size of the particle is positively charged and small in size, it will be able to enter the cell through translocation. This is made possible when a particle (cationic or anionic) comes close enough to the cell’s membrane to create a tiny hole for the uptake of the particles. Cationic agents use this membrane potential-driven translocation to enter the cell’s nucleus, showing better cellular uptake than their uncharged counterparts [71]. 

The cell nucleus membrane is thought to carry an additional negative potential that can be exploited to allow cargo uptake. For this reason, cationic agents have a greater nuclear localisation rate than anionic-charged particles [72]. Liposome complexes more commonly undergo clathrin-mediated endocytosis, caveolae-mediated endocytosis and micropinocytosis when it comes to cellular uptake in most cells, especially cancer cells, except phagocytes [31]. This is due to their bigger particle size, which makes it impossible to go through direct translocation. According to live cell imaging, cationic liposomes were observed to favour entering the cell through micropinocytosis [56].

Clathrin-mediated endocytosis is a means for cells to obtain nutrients, plasma membrane components, and ions from a clathrin-rich area at the plasma membrane near the cell surface [71]. Clathrin, classified as a protein, has a three-legged structure known as the triskelion that will co-assemble with other proteins in a cell to form a membrane curvature, which then leads to vesicle budding. Very often, particles (<250 nm) entering via the endosome will undergo lysosome degradation when they go through this route, but positively charged particles mostly favour clathrin-mediated endocytosis [71]. When positively charged particles enter the extracellular medium, they will reach the cell membrane by diffusion. Once they are close to the cell membrane, these positively charged particles will be attracted to the negatively charged clathrin. With more positive particles attached to clathrin on the surface of the cells, it will alter the local electric field, and these particles will be pushed further into the membrane surface and cause anchoring. The clathrin-mediated endocytosis process will be accelerated when the particle-cell plasma membrane contact area rises [72]. 

On the other hand, the caveolae-mediated endocytosis pathway is responsible for cell signalling, transcytosis, regulation of lipids, fatty acids, membrane proteins, and membrane tension. Particles (50–400 nm) that enter the cell through this pathway sometimes tend to avoid endosomal entrapment and, subsequently, lysosomal degradation. As particles enter the cell without entrapment, this will better enable the cargo to be delivered into the cell, successfully avoiding lysosomal degradation [73]. Since particles entering the cell by caveolin-dependent mechanisms can avoid lysosome degradation, pathogens such as viruses prefer to enter the cells through this pathway. 

Macropinocytosis works slightly differently compared to caveolae-mediated endocytosis and clathrin-mediated endocytosis, as it does not involve lipid rafts or other proteins for endocytosis. Cytoskeleton rearrangement will occur in the cell, and membrane extension or ruffles are formed when engulfing particles outside the cell, thus creating large vesicles (200–2000 nm) and allowing extracellular fluid to enter the cell. This pathway can take up larger particles that cannot go through either of the previously mentioned alternative pathways [71]. Strategies in formulating a cationic liposome will have to understand the pathway preferred for the right targeted uptake strategy, which can be achieved by creating vesicles with the right size that favour certain cell entering pathways.

### 4.4. Transfection Ability of Cationic Liposomes 

Cationic lipids with primary amines have a lower transfection efficiency than those with secondary or tertiary amines. As the density of positive charge in the formulation increases, this leads to poor DNA separation, a higher aggregation rate of the liposome complex, and thus a decrease in gene transfection efficiency [15]. It can be said that the transfection efficiency of a cationic liposome decreases with increasing alkyl chain length, type, and saturation, whereby the shorter alkyl chain is preferred [55]. According to Kumar et al. [74] findings, longer alkyl chains are more rigid because of their higher hydrophobicity and packing orders, but shorter alkyl chains have better membrane fluidity [74]. It has been proven that cationic lipids with shorter alkyl chains have better transfection efficiency under in vitro testing [35]. Creating a liposome with the optimum chain length for the best transfection efficiency is challenging. Creating a balance between hydrophobic and hydrophilic chain length, especially with the utilisation of a spacer, is still possible to formulate an efficient liposome. Chain length and spacers may contribute to bilayer membrane flexibility, and large vesicles can easily be converted to tiny vesicles by adding external energy, such as sonication [75]. Using a spacer helps unravel the lipid bilayers, allowing more accessible release of the cargo and forming smaller particles. Unique spacers are tailored to be sensitive to different environments (pH), so the release control can occur at strategic locations to increase transfection possibility. Thus, the molecular structure of the lipids that form liposomes greatly influences their size. 

Researchers in Norio Iijima, Department of Anatomy and Neurobiology, Nippon Medical School Japan, conducted research comparing the efficiency of cationic and anionic liposomes [76]. They found cationic liposomes have a more rapid and better transfection efficiency than anionic liposomes in cultured cells. Both anion and cation liposomes have different processing patterns and intracellular activity, which leads to the variation in transfection efficiency. When certain cationic liposomes are administered into the cells, their positive surface charge will undergo electrostatic interaction with the negatively charged cell membrane. This will lead to cell membrane disturbance whereby liposomes collapse into the cell and release their cargo quickly and directly into the cytosol, enabling cationic liposomes to bypass the endosome-lysosome system [76]. Therefore, cationic liposomes will be a more favoured transfection technique for transfecting cells than anionic liposomes.

### 4.5. Disadvantages of Cationic Liposomes

Although cationic liposomes seem like the ideal delivery system due to various factors, many things still need to be understood for this system to be a safe and effective breast cancer treatment. The limiting factor of using cationic liposomes in gene therapy is their instability upon storage and inactivity in the presence of serums [25,50,77]. In addition, the cytotoxicity of cationic liposomes is another factor that needs to be worked on and overcome. Cytotoxicity, in particular, will cause uncontrollable massive cell deaths in the host in numerous areas exposed to the system. For instance, pulmonary cells and arterial cell walls will be affected, and the normal functionality of a biological environment will cease. Then, pulmonary toxicity caused by the production of reactive oxygen intermediates will occur caused by liposome-induced oxidative burst, especially in lung cells, when introduced in vitro [78]. 

Furthermore, the formation of aggregates when injected into a host and interacting with circulating blood is another factor that will cause cytotoxicity. Phagocytic macrophage toxicity is also one of the causes of the cytotoxicity of a delivery strategy [50,79]. Compared to neutral and negatively charged liposomes, cationic liposomes are more readily taken up by phagocytic cells. This will then trigger the formation of reactive oxygen species (superoxide and hydroxyl radicals, nitric oxide, singlet oxygen, nitrogen dioxide, and peroxynitrite), which damages organelles and promotes higher intracellular sodium ion levels, which will lead to cytotoxicity and cell apoptosis [61]. 

Although the cytotoxicity effect of cationic liposomes is undeniable, the liposome’s formulation can be modified to overcome it. For instance, the addition of polymers or co-polymers such as polyethyleneimine (PEI), chitosan, poly-L-lysine (PLL), spermidine or PEG into a formulation can shield the positively charged lipid, thus reducing the effective charge of the liposome [69]. Adding cholesterol into the formulation has also been proven to reduce the charge of the liposome by having fewer cationic lipids in the formulation [80]. By reducing the charge, the cytotoxicity of cationic liposomes can be overcome. 

### 4.6. Cationic Liposome Formulations for Breast Cancer Therapy Strategy

Drug delivery systems have been employed to increase anticancer drug characteristics and release [26,81], but the major challenge is when drug-resistant tumours emerge. Thus, a strategy of combining drug and gene therapy has been studied. Bulbake et al. [82] used a cationic liposome formulation combining DOTAP and cholesterol as a form of a carrier for both chemotherapeutic drugs and genes as a sort of combined treatment for breast cancer cells (MCF-7 and MDA-MB-231). According to their findings, combining a medication (paclitaxel) with a therapeutic gene siRNA (si-Plk1) looks to be a viable technique for generating a new breast cancer treatment. This is because this method outperformed the single therapy of paclitaxel or siPlk1 by 35–60% [82]. Another study combined an anti-cancer medication (docetaxel) with a silencing gene (shRNA) to overcome drug-resistant issues. Cationic liposome formulations, including DOPE, DOTAP, PC, and cholesterol lipids, were evaluated on MCF-7 and MDA-MB-231 breast cancer cell lines and in vitro testing [83]. In vitro studies revealed that the combination vector did elicit more cell apoptosis than the single vector technique. In vivo, research revealed that combination therapy reduced tumour size by 26% and 43%, compared to liposome with docetaxel and liposome with shRNA.

Since peptides can attach to receptor proteins in cells or on their surfaces, they can be integrated into liposome formulations and utilised to target or guide the liposomes to specific target receptors or cells. A delivery system combining DOPE and DOTAP liposomes with a well-defined synthetic multifunctional peptide, DEN-K(GALA)-TAT-K(STR), was created and optimised for gene delivery to create an efficient gene delivery platform for breast cancer cells (MCF-7). To that end, a breast cancer-specific cell targeting peptide (CTP) was added to the peptide-based gene delivery system to promote cell-specific internalisation. Its efficacy in combination with a liposome was studied. When compared to the peptide or liposome formulation procedures alone, this formulation approach resulted in synergistic gains in gene expression and silencing. The system’s efficacy was established by successfully delivering B-cell lymphoma-2 (BCL-2) siRNA to MCF-7 cells, which resulted in near total suppression of the BCL-2 protein and inhibition of MCF-7 cell migration in a wound healing assay [84].

## 5. Anionic Liposomes

Lipids with a negative charge from the anionic phospholipid head groups form anionic liposomes. Anionic liposomes were not widely used in gene therapy in the early years due to their negatively charged properties, which repel the phosphate backbone of nucleic acid cargoes that are also negatively charged. This causes repulsive electrostatic forces between them and makes encapsulation of genetic material within the vesicle impossible [25,27]. Phosphatidic acid, phosphatidylglycerol, and phosphatidylserine, found naturally in cellular membranes, are negatively charged lipids used to synthesise anionic liposomes. Some of the commonly used anionic lipids to formulate liposomes are summarised in Table 6. The usage of anionic liposomes in gene therapy first started when Akhtar et al. [85] created a model studying the non-receptor-mediated transport of DNA or plasmid from the cell surface into the cell body. They have also been using anionic liposomes in transferring oligonucleotides into hippocampal neurons, inducing transgene expression, which was unsuccessful when using cationic liposomes [85,86]. Another study utilised anionic lipoplexes (anionic liposomes combined with calcium ions, Ca^2+^, encapsulating siRNA) for gene silencing in breast cancer cell culture, claiming that they were the first to discover the practical usage of anionic liposomes without using cationic lipids or polymers for gene silencing. As a result, the efficiency of this optimised combination was comparable to cationic lipoplexes (Lipofectamine 2000) [58]. Giulimondi et al. [51] created a lipoplex by combining anionic liposomes with cations (e.g., Ca^2+^, Mg^2+^, Fe^2+^, etc.) as a gene-delivery system with a similar approach to Kapoor and Burgess [51,58]. This was accomplished because the divalent cations could overcome the anionic liposome’s mutual electrostatic repulsion with the phosphate backbone of DNA.

Forssen and Tokes used anionic liposomes to encapsulate doxorubicin, an antitumor agent, which is impossible using neutral or cationic liposome systems. By encapsulating the cationic drug in anionic liposomes, cardiotoxicity was reduced with positive antitumor activity. Due to Forssen and Toke’s findings, anionic liposomes have been widely used in drug delivery [87]. This finding led to the development of Doxil, an example of a doxorubicin hydrochloride drug encapsulated in a polyethene glycol (PEG)-coated liposome (HSPC/CHOL/DSPE-mPEG) for intravenous administration. It is one of the drugs approved by the US Food and Drug Administration (FDA) that uses anionic liposomes to treat ovarian and breast cancer. Besides attaching PEG onto the liposome, Wang et al. [88] and his team used phosphatidyl ethanolamine (PE) to create an anionic liposome system with positively charged polymer polyethyleneimine (PEI) that is lower in cytotoxicity compared to cationic lipids [88]. This formulation was used to encapsulate the p21-siRNA-322 gene, which was made possible in the presence of positively charged PEI and hyaluronan (HA) attached to the surface of the liposome. Chen et al. [89] have discovered a p21-saRNA-322 gene that can activate the p21 gene in the cell to stop the proliferation of cancer cells [89].

### 5.1. Stability of Anionic Liposomes 

Anionic liposome systems are more stable in suspension and plasma media [31,84,85] because they cause fewer aggregations than neutral and positively charged liposomes. Anionic lipids such as 1,2-Dipalmitoyl-sn-glycero-3-phospho-rac-(1-glycerol) (DPPG) have a high phase transition temperature, which increases the rigidity of the particle’s membrane, thereby enabling it to inhibit the penetration of serum proteins and lipoproteins in serum. However, Bozzuto et al. [90] reported a different finding whereby negatively charged liposomes are less stable when administered into the bloodstream than cationic and neutral liposomes [90]. This is attributed to the electrostatic properties between the negative charge on the liposomes and the circulating proteins in the blood. This phenomenon will lead to rapid reticuloendothelial system absorption and cytotoxicity effects, which lead to pseudoallergy symptoms such as vasoconstriction, pulmonary hypertension, dyspnoea, and a reduction of platelets and leukocyte count. Therefore, until now, no treatment has used an anionic liposome as a carrier for treatments involving intravenous administration.

Although attaching polymers to the formulation with anionic lipids will be able to prevent electrostatic interaction in plasma or serum, stability remains an issue. Adding a polymer component into the formulation will alter the zeta potential, size, transfection efficiency, cytotoxicity, stability and uptake of the particles, making formulation complicated with many parameters that need to be considered. There have been efforts to incorporate PEI cationic polymer into their anionic liposome formulation. However, obtaining the proper ratio or balance has been challenging because too much polymer in a formulation will alter the transfection efficiency due to low nucleic acid compaction within the carrier and cause lower stability. When a higher molecular weight polymer or an increase in N/P ratio is used, the selectivity and efficacy of the carrier to transfer genes will be reduced [91]. Though this type of formulation shows promising results in vitro, it is not entirely successful when administered in vitro due to its low stability when stored or exposed to salt and serum within a biological system or environment [92,93]. To sum it up, anionic liposomes are more stable in a biological environment than cationic liposomes. However, modifications still need to be made to the anionic liposome formulation to overcome its electrostatic repulsion with the negatively charged nucleic acid for it to be a suitable carrier in gene therapy.

### 5.2. Cytotoxicity of Anionic Liposomes 

Cytotoxicity has always been the downfall of cationic liposomes, and due to this reason, many have ventured into investigating anionic liposomes. Anionic lipids show lower cytotoxicity compared with cationic liposomes, mainly due to anionic lipids’ being similar in composition to natural cell membranes. They have high immunotolerance with high cellular uptake. It also has the potential for high transfection efficiency due to escaping the endosomal pathway with direct fusion into the cell membrane and releasing its cargo into the cytosol. Despite all these good traits, using anionic lipids on a large industrial scale is highly impossible as it is very complex (relying heavily on immunolocalisation tests, which frequently fail due to membrane integrity losses), and its production cost is very high [93]. 

Kurosaki et al. [94], Hattori et al. [60], and Chen et al. [95] conducted research in which they encapsulated cationic liposomes with a negatively charged polymer and demonstrated lesser cytotoxicity. In this particular strategy, the cationic liposome-encapsulated nucleic acid. The toxicity level was significantly improved due to the shielding effect of the negatively charged polymer [60,94,95]. Researchers have also been trying to attach polymers such as PEI that are positively charged into anionic liposomes. Through this method, they can make the surface charge of the carrier positively charged. However, formulating PEI into an anionic liposome is challenging as different molecular weights of PEI will give different outcomes to the carrier cytotoxicity. According to Ewe et al. [92] and Jerzykiewicz et al. [93], certain low-molecular-weight linear PEIs have significant efficacy and minimal cytotoxicity to be successfully incorporated into liposome formulation compared to higher-molecular weight PEIs [92,93].

A different approach was also tried involving diluting the charge. An approach is similarly made for cationic liposomes. However, according to Nchinda et al. [96], just diluting the surface charge of a liposome formulation with a neutral lipid does not always reduce the cytotoxicity, as there were incidents where it increased the cytotoxicity [96]. Moreover, the amount of cholesterol used to form liposomes with the necessary properties must be carefully examined [97]. As maximising the action of cholesterol in a liposome formulation is already a challenge, the ratio is a crucial component that will affect liposome encapsulation efficiency and transfection. 

Furthermore, surface modification for lengthy circulation, stability in circulation, cytotoxicity, cellular uptake, and effective cargo delivery at the target location must be considered when formulating a liposome formulation [98]. Just tweaking the combination of lipids in a formulation itself may raise complications; attaching polymers into a formulation will be even more challenging and complicated. The qualities of liposome complexes with additional attachments on their surface must be evaluated experimentally to produce stable liposomal systems that can circulate in the blood for a more extended period to ensure the formula is stable enough for the treatment system to work. Over-modification of the liposomal surface with attachment should be avoided, and it should be able to interact freely with target cells [98]. For these reasons, many would prefer to formulate liposome formulations with cationic lipids, which is less complicated. With proper planning, modification, and a sound strategy, anionic liposomes can still be effective as a gene delivery system, though a lot more work needs to be performed.

### 5.3. Cellular Uptake of Anionic Liposomes

Anionic liposomes initiate a better endocytosis rate through macropinocytosis than cationic and neutral liposomes [56,99]. Anionic liposomes do not initiate electrostatic interaction but create an electrical repulsion with cell membranes. The negatively charged liposomes have a lower chance of undergoing phagocytosis by macrophages because of their similarities with the host’s cell membrane, leading to slower kinetics of clearance from the host body, thus, better retention in the biological system [100]. According to Kapoor M. and Burgess D.’s findings, a similar uptake pathway to cationic liposomes can still be achieved by attaching cationic ions to their surface. Their results have proven that anionic liposomes with Ca^2+^ cations could enter cancer cells through clathrin and caveolae-independent pathways similar to cationic liposomes [58]. Not only does it behave like cationic liposomes after this modification, but it was also proven to be less cytotoxic than its counterpart with their optimum formulation. Furthermore, an abundance of calcium ion concentration will result in severe cytotoxicity; thus, a balance needs to be achieved to ensure safe and optimal liposome performance. However, there has been an increasing interest in developing anionic liposomes as carriers for transdermal drug delivery due to their enhanced penetration properties through the skin. Histological studies revealed that the anionic liposomes diffused into the dermis and the lower portion of hair follicles through the stratum corneum and follicles more effectively than cationic liposomes. Therefore, the rapid penetration of negatively charged liposomes would contribute to the increased permeation of cargo through the skin [90,101]. Anionic liposomes demonstrated substantial intracellular uptake via intradermal penetration but were not as effective as cationic liposomes. Anionic liposomes did not cause significant skin irritation, as cationic liposomes do, and they have the potential for efficacy and safety. As a result, anionic liposome formulations are ideal for use as a medication delivery system rather than a gene delivery carrier, as anionic liposomes have a superior penetrating ability with less toxicity, which cationic liposomes do not have. Furthermore, anionic liposome formulation with cationic ions can boost encapsulation efficiency; nonetheless, more study is required to develop a viable non-viral gene carrier with efficient cellular uptake.

### 5.4. Transfection Ability of Anionic Liposomes 

The effectiveness of transfection of anionic lipoplexes was comparable to that of cationic liposomes, but their toxicity was significantly lower. Anionic liposomes have shown the ability to release cargo in their hollow vesicles faster than neutral liposomes [102]. Furthermore, anionic liposomes have also shown potential to be used as a delivery system, especially when delivering drugs to the extravascular site, which is not achievable when cationic liposomes are used [38]. However, formulating anionic liposomes as a carrier for genes is still possible. Modification can be performed to overcome the repulsive force of the same charged surface between the cell membrane, the potential therapeutic gene and the anionic liposomes. Some strategies being tried involve attaching cations onto the surface of the anionic liposomes to imitate the surface of cationic liposomes. It was reported that among all of the divalent cations tested, Ca^2+^ has the highest transfection efficiency due to its higher nucleic acid binding affinity [103,104]. The concentration of Ca^2+^ is crucial to produce an efficient gene delivery system with low toxicity. If used in excess, it will form a lipoplex with a particle size of more than 500 nm, which exceeds the ideal transfection particle size of 200 nm that favours clathrin-mediated uptake. Ca^2+^ can facilitate nucleic acid transfection but will lead to precipitation, resulting in an unfavourable transfection rate [50]. Furthermore, this complex (Ca^2+^ and nucleic acid) shows irregular transfection due to the high calcium ion inside the cell, causing interactions with the lipid of the cell membrane, leading to lipid redistribution and contributing towards unfavourable toxicity.

The cellular transfection of cationic and anionic liposomes has been extensively compared to identify the more favourable system. It was found that anionic and cationic liposomes enter the cell through very different and distinct pathways. Negatively charged liposomes will tend to be engulfed by the cell (endocytosis) instead of fusing into the cell as most tiny cationic nanoparticles do. Although bigger-sized cationic liposomes and anionic liposomes enter the cell through endocytosis, their subsequent pathways are very different. Cationic liposomes can initiate a proton sponge effect that will efficiently escape the endosome-lysosome compartment, but anionic liposomes, on the other hand, prefer the endosome-lysosome pathway. Engulfed anionic liposomes will immediately be transferred into the endosome-lysosome system with a higher tendency to be digested instead of entering into the cytosol directly like their positively charged counterpart. This endosome-lysosome pathway will allow lysosome enzymes to digest the liposome layer and release the cargo transported into the cell. Therefore, due to this mechanism, it can be said that anionic liposomes are more suitable for drug delivery systems as a carrier for lysosome-resistant reagents and lysosome-targeted drugs. Due to this, the nucleic acid transfection system using anionic liposomes as their carrier is not suitable without prior modifications or adjustments [76], as the lysosome enzyme will digest nucleic acids.

### 5.5. Disadvantages of Anionic Liposomes 

pH sensitivity has been the main disadvantage for anionic formulations in gene transfer compared to their cationic counterparts [88]. Other than that, during the synthesis of anionic liposomes, high concentrations of lipids are required to increase the success of anionic lipoplex formation via passive encapsulation of nucleic acid. Encapsulating nucleic acids can only be made possible when the surface of the nucleic acid is attached to cationic components such as cationic polymers or ions (Ca^2+^). Additional polymers or ions attached to the cargo will increase the size, requiring more lipid molecules to encapsulate the nucleic acid within the carrier’s core fully. Furthermore, anionic liposomes have poor encapsulation efficiency for failing to condense nucleic acid, which does not occur with their positively charged counterpart. This is mainly due to the repulsive electrostatic interaction between two anionic substances [25,43,90]. As a result, high amounts of empty liposomes are created, and to overcome this, repeated protocols such as freeze-thawing cycles and sonication need to be performed. These procedures will tend to lead to nucleic acid damage and degradation [105]. Of course, attaching cationic polymers or ions can overcome the electrostatic repulsion between the anionic liposomes and the nucleic acid, but just formulating a liposome that is stable with low cytotoxicity, high transfection efficiency, and cellular uptake is already a challenge without the additional attachments or modifications. 

### 5.6. Anionic Liposome Formulations for Breast Cancer Therapy Strategy

Another strategy was discovered that enables anionic liposome to be used as a non-viral vector to encapsulate potential therapeutic genes overcoming electrostatic repulsion between anionic liposome and negatively charged nucleic acid. Creating liposomes with asymmetric charge distributions can have different net charges of lipids on their outer and inner leaflets [90]. The liposomes consist of zwitterionic lipids and cholesterol combined with either cationic or anionic lipids. This method allows encapsulation of anionic-cargo-like nucleic acid using anionic lipid without electrostatic repulsion as the inner leaflet consists of neutral lipids. Regardless of the identity of the outer leaflet lipids, interior leaflet lipids can be chosen to enhance the liposomal loading of charged cargo. This indicates that it is possible to independently modify outer-leaflet lipids to improve lipid vesicle bioavailability, biodistribution, entrapment efficiency, and slow leakage features, which makes liposome formulation modification endless possibilities.

## 6. Neutral Liposomes

Neutral liposomes are typically formulated using neutral or zwitterionic (can be negatively or positively charged or remain neutral in charge at physiological pH and thus will change according to the environment) lipids such as phosphatidylcholine, cholesterol, and EPC are neutral. At the same time, DSPE (1,2-distearoylsn-glycero-3-phosphoethanolamine), DPPC (1,2-dipalmitoyl-sn-glycero-3-phosphocholine) and DOPE (1,2-dioleoyl-sn-glycero-3-phosphoethanolamine) are zwitterionic [51]. Table 7 summarises the commonly used neutral lipids used in formulating liposomes. Bangham et al. [24] formulated the first neutral liposomes using a combination of lecithin and cholesterol. However, neutral liposomes are physically less stable because their aggregation properties are lower in colloidal stability. This is due to the lack of charge that precludes the creation of a stable aggregation with a negatively charged nucleic acid. This is absent in charged liposomes, whereby the charged surface will induce electrostatic repulsion that prevents particles from aggregating [24,59,90]. Moreover, natural electrostatic attraction between positively (cationic lipid) and negatively charged particles (nucleic acid) made from forming complexes is more efficient than neutral lipids. However, their surface charge is simpler to modify to modulate the diffusion of different cation and anion substances. For instance, proteins can be introduced into their structure, and their composition can be altered to replicate the features of a wide range of natural membranes. Furthermore, it was anticipated that if liposomes could contain proteins, enzymes, medicines, or nucleic acids, an essential step toward a full in vitro reproduction of living system membranes would be obtained [59]. 

Neutral liposomes exhibit a longer circulation time with lower cytotoxicity than cationic liposomes because they do not interact with negatively charged proteins in serum and blood. Furthermore, neutral liposomes were shown to be primarily present in tumours and sites of inflammation because it is easier to apply active targeting strategies to neutral liposomes than cationic liposomes as there are nonspecific electrostatic interactions. Moreover, neutral liposomes do not interact significantly with cells, which causes non-targeted cargo release from the liposomes, as the interaction between cells and liposomes can only occur when the electrostatic interactions from a charged surface are present [90]. 

Successful encapsulation by neutral liposomes can be performed by using neutral lipids added with ethanol and calcium chloride into the aqueous mixture with a nucleic acid to form a lipoplex. DOPE is one of the widely used zwitterionic lipids used in formulating liposomes, but there is a risk of aggregation due to low colloidal stability. According to Krasnici et al., 2003 findings, neutral liposomes can encapsulate anthracycline drugs such as doxorubicin (DOX), epirubicin (EPI), daunorubicin (DAU), and idarubicin (IDA) that are cationic charged [106]. They produce complexes with better stability and reduce cargo leakage during circulation before reaching the targeted site. This enables it to be delivered to tumour sites without showing side effects on the host. Because cationic medicines and nucleic acids are both positively charged, it is reasonable to assume that the same liposome formulation can be employed as a gene carrier without major modification in their formulation. 

Neutral liposomes also have a delayed clearance in vitro compared to cationic liposomes [55]. Unlike anionic and cationic liposomes, once a neutral liposome is administered into a host, it will undergo rapid clearance. Anionic liposomes are identified as foreign particles that eventually end up in the reticuloendothelial system [90]. As for cationic liposomes will tend to be attracted to plasma proteins, forming a corona layer on their surface, and then be coated with antibodies, which will then be ingested by phagocytes [107]. From these findings, it can be said that formulating a functional liposome with only a single type of lipid is not possible, considering the limitations. However, a combination of different types of lipids can alter the characteristics and thus make the carrier more functional. Therefore, neutral lipids are usually utilised mainly as helper lipids in anionic or cationic liposome formulations or neutral vesicles with charged ions for a fully functional delivery system.

### 6.1. Stability of Neutral Liposomes 

Neutrally charged liposomes have lower colloidal stability when compared with charged liposomes. This is highly attributed to the lack of electrostatic repulsive forces that prevent aggregation [55]. In order to compensate for the lack of charge, positively charged ions or molecules were introduced into neutral liposome formulations to give them a distinctive charge on their surface to improve functionality. Chitosan, a positively charged molecule, is one of these options used to coat neutral liposomes made from cholesterol or phosphatidylcholine to increase their stability. Channarong et al. [108] formulated this by delivering a nucleic acid vaccine for Peyer’s patch targeting [108]. Usually, improved stability was achieved by utilising cholesterol in liposome formulation. This was attributed to the improved rigidity enabling a denser assembly of phospholipids. This prevents aggregation and closely packed structures from infiltrating or attracting serum proteins and improves blood circulation time [109]. 

Researchers have also tried to reduce the multivalent positive charged lipids by adding cholesterol into the synthesis of liposomes as a helper lipid. As a result, they form more stable complexes and work better during in vitro administration. Adding cholesterol into the synthesis of cationic liposomes was able to increase the concentration of the starting cationic lipids used, thereby increasing the amount of nucleic acid to be encapsulated in the formulation without altering the stability of the liposome and cargo complexes [55]. Without cholesterol in the formulation, charged particles will aggregate due to low electrostatic repulsion and form precipitates, contributing to the increase in particle size. In addition, it was also discovered that adding cholesterol into the formulation of liposomes could reduce the permeability and increase their stability. This is because cholesterol could pack denser phospholipids, thereby stopping liposomes from destabilising [108]. Therefore, neutral lipids are mainly used as helper lipids and stabilisers in liposome formulations rather than as the primary component in designing a non-viral carrier for gene therapy.

### 6.2. Cytotoxicity of Neutral Liposomes 

Many claims that neutral liposomes are nontoxic or have very low toxicity, as neutral lipids can be found naturally in cells [61]. Neutral lipids are mainly derived from the cell membrane, making them non-toxic and stable in blood circulation as they will not interact with proteins present in serum and blood like other charged carriers do [90]. Pisani et al. [59] compared cationic and neutral liposomes for their serum stability and cytotoxicity. They found that neutral liposomes show better stability when compared to cationic lipids due to the absence of charge that tends to interact with salt and other particles in the serum and blood, which will then lead to cytotoxicity [59]. 

Neutral liposomes can be used as carrier vesicles, with some modification performed to the surface of the vesicles to favour encapsulating nucleic acid and transfection. It is a great candidate as it shows excellent stability, considerably no toxicity, and does not undergo rapid clearance. Moreover, it will not be detected as a foreign particle and will go through opsonisation. Neutral lipids can also improve cationic or anionic liposome formulation, acting as helper lipids for better optimal performance in transfecting the cell and reducing cytotoxicity. Helper lipids such as 1,2-dioleoyl-sn-glycero-3-phosphocholine (DOPC), 1,2-dioleoyl-sn-glycero-3-phosphoethanolamine (DOPE), and cholesterol have been used in formulations to help improve transfection efficiency in a charged liposome formulation. This is mainly due to neutral lipids enabling the carrier to fuse readily and destabilise the bilayer membranes, making it easier for the cargo to escape from the endosomes inside the cell and helping with cellular uptake via penetrating through the cell membrane [59]. 

Neutral lipids such as cholesterol also act as linkers to attach other components such as polymers and proteins. Zwitterionic lipids such as DSPE and DOPE are usually used for attaching polymers and proteins with covalent binding to the surface of the liposomes. Most will opt for the covalent binding attachment strategy over non-covalent binding as the linkage formed by covalent binding is more stable and repeatable compared to the non-covalent binding method [110].

### 6.3. Cellular Uptake of Neutral Liposomes 

Liposome formulations with neutral lipids have better cellular uptake than those without them. Take cholesterol, for instance; it can go through a specific metabolic pathway for uptake into the cell involving specific receptors initiating the fission of liposomes to the cell membrane. Based on Kang et al. [56] findings via live cell imaging, neutral liposomes enter the cell through caveolae-mediated endocytosis, which makes high cellular uptake possible as this pathway will be able to allow neutral liposomes to escape lysosome degradation in the cell effectively [56]. Through this pathway, a cytosolic caveolar vesicle is formed by the fission of the caveolae from the membrane, which is mediated by the GTPase dynamin. This vesicle forms within the cell (caveosome) and does not contain an enzymatic degrading cocktail that is present in the lysosome and will be transported to the endoplasmic reticulum. Unlike clathrin-mediated endocytosis, where particles will end up being degraded by the lysosome, considering it is the only pathway. Caveolae-mediated endocytosis has an alternative pathway triggered by ligands such as folic acid, albumin, and cholesterol on the particles enabling them to escape lysosome degradation. Many pathogens use this route to avoid degradation by lysosomal enzymes [111]. 

Zwitterionic lipids such as DOPE, DPPC, and DSPC have a different strategy where they can go through endosomal escape to prevent cargo degradation and assure successful cytosolic delivery of the cargo. Since zwitterionic lipids are pH sensitive, zwitterionic lipids undergo changes at a lower pH (in the cell cytosol), from lamellar to inverted micelle structures, which trigger the union of the liposomal and endosomal membranes, thus destabilising the endosomes and leading to cargo release [111]. However, this strategy will be a challenge when liposome formulation with zwitterionic lipids is introduced in vitro due to the stability of the liposome in the presence of serum.

From these, neutral liposomes and charged liposomes have very different cellular uptake pathways. Other than that, neutral liposomes or zwitterionic liposomes are less prone to protein binding, which can lower the rate of cellular uptake when compared with other charged liposomes [112].

### 6.4. Transfection Ability of Neutral Liposomes 

Cationic carriers have serious drawbacks that affect their transfection efficiencies, such as cytotoxicity towards cells, cell shrinking, inhibition of the protein kinase C, and limited stability of their complexes with plasmid DNA in serum. These reasons are responsible for the current restriction on the extensive commercial use of cationic liposomes. Thus, another alternative that is less toxic to the cell in the form of neutral liposomes is being heavily investigated and considered. Transfection can only occur when the nucleic acid is meant to be transfected into the nucleus or dissociation happens in the nucleus [31]. Therefore, unpacking the liposome with nucleic acid complexes is crucial for releasing nucleic acid, and the release should occur as near the nucleus as possible. If released far from the nucleus, the nucleic acid will find it challenging to move into the nucleus, with a high chance of being degraded. 

Cellular transfection has proven to be a complex technique, comprising numerous steps, slowing the development of efficient and safe therapeutic applications. Pure neutral liposomes such as dioleoylphosphatidylcholine (DOPC) have very low transfection efficiency in delivering nucleic acid (green Lantern), even with the incorporation of cationic ions such as Mg^2+^ and Ca^2+^ When a combination of two different neutral lipids, DOPC and 2-dioleoyl-sn-glycero-3-phosphoethanolamine-N-hexanoylamine, was used in the formulation, the transfection efficiency improved by six folds [59,113]. Comparing two different zwitterionic lipids incorporated into cationic liposomes found that one is more efficient in improving transfection than the other. When four types of lipids were combined (DOTAP/DOPC/DOPE/DC-Chol), the transfection efficiency was greater than when DOTAP/DOPC and DOPE/DC-Chol were used separately [114]. This result shows the possibility of neutral liposomes having the potential as gene carriers with the appropriate conditions and the right strategy used for their formulations.

Cholesterol is naturally present and distributed in the membranes of eukaryotic cells and plasma membranes. A group of researchers from the University of Chicago discovered that increasing cholesterol concentration in human fibroblast cell culture triggers the extra cholesterol’s movement into the cell’s intracellular compartments [115]. Since then, cholesterol has been used as a major lipid and helper lipid in formulating liposomes for gene delivery as it shows better advantages over other neutral lipids. It has been reported that adding cholesterol into cationic liposomes can increase liposomes’ efficiency compared to formulations without it. Liposome formulations with cholesterol showed more efficient escape from the endosome, subsequently leading to better transfection efficiency [31,73]. The efficiency of a liposome’s formulation will show different transfection efficiencies in different types of cell lines, different types of cargo, and the location it needs to be transfected.

### 6.5. Neutral Liposome Formulations for Breast Cancer Therapy Strategy

Neutral liposome formulation cannot perform optimally without modifications or attachments. There were different approaches and modifications performed to develop a functional liposome-based gene therapy system as a form of gene therapy treatment for breast cancer. Hyaluronic acid (HA) was included in liposome formulation to increase the targetability of breast cancer tumours since HA binds to CD44, which is overexpressed in tumour cells [89,116]. Hayward et al. [116] carried out an innovative work whereby they administered the miR-125a-5p gene to HER2-positive metastatic breast cancer cells (21MT-1) using a neutral-charged lipid-based system made up of L-α-phosphatidylcholine (PC), palmitoyl-sn-glycero-3-phopshoethanolamine (DPPE) and cholesterol (CHOL) coated with hyaluronic acid. This liposome formulation was used to encapsulate the miR-125a-5p gene. It significantly inhibited HER2 expression, cell proliferation, and migration in the 21MT-1 cell line via the PI3K/AKT and MAPK signalling pathways [116]. This study is similar to Chen et al. [89] liposome formulation targeting method, which uses HA as a targeting ligand in their liposome formulation, which has been discussed previously [89].

Aptamers potentially bind to a wide range of molecular targets with high affinity and specificity; therefore, conjugating aptamers to liposomes improves active targeting. A liposomal-based siRNA delivery system with a core made of siRNA:protamine complex and a shell designed for active targeting of CD44-expressing cells employing an anti-CD44 aptamer (called Apt1) as targeting ligand was investigated in this study. siRNA was encapsulated within a liposome composed of DPPC, cholesterol, and DSPE-PEG with Apt1 aptamers containing 2′-F-pyrimidines. This novel non-cationic method was tested in vitro and in vitro for the silencing of the luciferase reporter gene (luc2) in a triple-negative breast cancer model. The formulation inhibited luc2 in vitro with aptamer functionalised liposomes loaded with siRNA and prolonged inhibition in vitro [117].

A different trial used an antibody against an overly-expressed heparin-binding epidermal growth factor in the MDA-MB-231 cell line and TNBC breast cancer tumour. These targeting antibodies were conjugated to anionic liposome formulations containing dimyristoylphosphoglycerol (DMPG), distearoylphosphatidylethanolamine-polyethyleneglycol (DSPE-PEG) 5000, and maleimide-conjugated DSPE-PEG5000 (DSPE-PEG-mal), DOPE, and cholesterol encapsulating therapeutic siRNA to induce gene silencing. The results revealed that this formulation had long-term blood circulation with tumour accumulation ability and inhibited PLK1 protein production and tumour growth [118]. Liu et al. [119] investigated a molecule-targeted and synthetic lethality-based siRNA therapy for TNBC treatment that employs cationic lipid with poly(ethylene glycol)-b-poly(D,L-lactide) nanoparticles as the siRNA carriers. The delivery of siRNA targeting cyclin-dependent kinase 1 (CDK1) caused cell viability to decrease due to cell apoptosis through RNAi-mediated CDK1 expression inhibition. This was observed only in c-Myc overexpressed TNBC cells (SUM149 and BT549) but not in normal mammary epithelial cells (MCF 10A), indicating that the synthetic lethality involving c-Myc and CDK1 was specific only to TNBC cells. In in vitro testing, the treatment suppresses tumour growth in mice bearing SUM149 and BT549 xenografts while causing no systemic toxicity or activating the innate immune response, implying the therapeutic potential for c-Myc overexpressed triple-negative breast cancer [119].

## 7. Future Perspective

Liposomes have been increasingly used in medication administration since 1994, when the FDA approved the first PEGylated liposome-based nano drug delivery system, Doxil^®^ [81,120]. Although encapsulating medications with liposomes reduces toxicity and allows for increased therapeutic administration to breast cancer patients, the overall performance is not preferred as patients still experience side effects such as cardiotoxicity and alopecia. As a form of carrier, liposomes meets all of the criteria for an appropriate delivery vehicle, including being biodegradable, biocompatible, and stable. When administered to a patient, liposomes can protect cargo from degradation and reduce nonspecific toxicity, and they are simple to produce and design for target-specific delivery [26]. Although liposomes have performed reasonably well as a drug delivery vehicle, no liposome formulation utilised as a gene carrier has been established as a form of treatment for breast cancer. Table 8 summarises liposome formulations evaluated on various forms of gene therapy strategy for breast cancer that have been published. These successful formulations for different types of breast cancer will provide a better understanding of how to produce an optimal liposome formulation. This will narrow down the possible formulations that are serum stable, have minimal cytotoxicity, have high cellular uptake, and have a high transfection efficiency. This review examines several approaches and strategies to create efficient liposomal-based gene carriers for breast cancer treatment.

These investigations suggest that cationic liposomes are preferred over anionic and neutral liposomes as their liposome base formulation. It is easier to put together with minor modifications to formulate a functional non-viral gene carrier. Neutral liposomes also showed potential due to their robust modification ability by utilising different attachments such as polymers, aptamers, peptides, antibodies, and overly expressed receptor-binding ligands such as hyaluronic acid. Combination strategies reported have proven that liposome formulations are adaptable and can be easily modified into the desired treatment to treat breast cancer. However, to be able to formulate the right combination of lipids in a liposome formulation is already tedious; major surface modification and attachments will make formulating a functional non-viral gene carrier even more challenging. More liposome-based non-viral gene carriers are currently in the design stage and may progress to preclinical development for cancer therapy in the future. For accurate clinical and commercial translation, more use of targeting ligands specific for overexpressed receptors at metastatic sites, a combination of treatments using drug treatment and gene therapy as an example, followed by treatment system optimisation, is required to make them adaptable in vivo to the complex challenges. Despite the challenge, liposomes do have the potential to overcome current clinical obstacles and shape the future treatment scenario by reducing the suffering associated with traditional chemotherapy and surgery. Despite the numerous approved and ongoing clinical trials, significant efforts are still required to overcome the physiological barriers impeding the liposome gene delivery systems as a treatment for breast cancer. Liposome-based gene therapy treatment strategies will undoubtedly be a pillar for future breakthroughs and progress in personalised medicine, paving the way for increased collaboration with experts in clinical oncology, pharmacokinetics, toxicology, immunology, and nanotechnology and opening doors to other better alternatives cancer treatments and even other genetically related diseases.

## 8. Summary

Generally, cationic liposomes, with their positively charged properties, will create an electrostatic interaction with the nucleic acid and cell membranes that are naturally anionic or negatively charged. The cytotoxicity and stability issues of the positively charged liposomes can be overcome by modifying their surface membrane or lipid combinations. Its intracellular activity that incorporates releasing its cargo into the cytosol instead of transporting it into the endosome makes cationic liposomes a potential vehicle for carrying genes for a targeted gene therapy strategy with high transfection efficiency. Therefore, liposomes are seen as a feasible non-viral nanoparticle strategy used in gene therapy and as a means of transporting drugs in drug delivery systems. The liposomes’ potential in the said treatment methods is due to their flexibility in being modified to have the most optimal functional system with desired traits as a vehicle in these treatments, either by using the liposome at its basic liposome formulation or by adding other substances such as polymers, peptides, proteins, polynucleotides, polycations, ligands, or receptors, which can also be used to overcome the cytotoxicity and increase the transfection efficiency of the liposomes.

Anionic liposomes were shown to have lower cytotoxicity and high stability in the biological environment than cationic liposomes. Still, the transfection of bare anionic liposomes into the cell is impossible due to the repulsive force of their negatively charged surface with the negatively charged nucleic acids and cell membranes. Thus, modifications are needed, especially on the surface of the anionic liposomes. Modifications can be performed by attaching cationic molecules onto the surface of the anionic liposomes, but it comes at a cost. The attachment of cationic molecules to the surface of anionic liposomes raises cation toxicity. Thus, a cation concentration threshold must be established and not exceeded to prevent the unfavourable outcomes that cation toxicity can cause, such as aggregations. With all these parameters to be considered, formulating a good anionic liposome vehicle can be more tedious and difficult to control.

Furthermore, anionic liposomes have a different intercellular uptake pathway when compared with cationic liposomes. Most of the time, anionic liposomes will end up in the endosomal-lysosome system, causing the cargo to be digested in the cells. Therefore, anionic liposomes are more suitable and less complex to be formulated for drug delivery systems, considering not so much in gene delivery systems. However, their success is still questionable in gene delivery strategies.

Neutral liposomes are the least cytotoxic systems due to their structure being similar to the cell membrane’s composition and lacking any particular component that instigates cytotoxic mechanisms. However, neutral liposomes will make targeting difficult, so ligands or lipid modification attachment is needed to achieve such a function. Therefore, similarly to anionic liposomes, neutral liposomes are more commonly used in drug delivery and are not widely used in gene therapy as there is no surface charge to aid in the encapsulation of the negatively charged nucleic acid. However, in recent years, efforts have been made to utilise neutral liposomes as gene carriers using various strategies and approaches, particularly for gene therapy. Most of the time, modifications in neutral liposomes are performed by attaching cations (Ca^2+^, Mg^2+^) and positively charged polymers onto the surface of the liposome to compensate for the absence of charge, improving the encapsulation efficiency and cellular uptake but without the cytotoxicity that is generally contributed by the highly positive charge of cationic liposomes. Polymer attachments to the liposomes inhibit liposome clearance by neutralising the liposome’s surface charge, which protects it from the attachment of proteins in serums and shields the charge of a charged lipid formulation. Neutral lipids are vital as supporting components in cationic and anionic liposome formulations by acting as helper lipids for the formulation. Incorporating neutral lipids into formulations of charged liposomes will reduce the charge of the overall liposome, leading to lower cytotoxicity, better cellular uptake and transfection efficiency. Referring to the summary of the review shown in Table 9, it can be said that the lipid most suitable to be formulated as a carrier for gene therapy specifically for treating breast cancer is a cationic, neutral lipid, followed by anionic lipid. There are still many gaps of unfilled information on formulating the most optimum liposome as a gene carrier, and many discoveries are deemed essential but have yet to be made.

Based on the comparison of cationic, anionic, and neutral liposome formulations, it can be concluded that the development of a potential breast cancer gene therapy system will benefit the most by utilising cationic liposomes as its delivery system base because they have a greater capacity to encapsulate nucleic acid, show great potential due to their positive surface charge affecting intracellular activity, and flexibility in modification to develop a more favourable gene therapy-based treatment. More research is needed to determine which lipid combinations can produce the best formulation for gene therapy that is stable, low in cytotoxicity, high in cellular uptake, and high in transfection efficiency. Moreover, developing an optimal liposome formulation base with optimal functionality will make studies of polymers, aptamers, peptides, antibodies, and ligand attachment easier to create a therapy system.

## Figures and Tables

**Table 1:** Potential factors that increase the chances of obtaining breast cancer with examples.

Factors	Examples
Heredity and genetic factors	Family history and breast cancer-causing gene mutation, pathogenic mutations in cancer-predisposition genes, and common single-nucleotide polymorphisms linked to breast cancer
Menstruation-related	Age of menarche and later age of menopause
Reproduction-related	Nulliparous, postponement of having firstborn, low rate of reproduction, and low rate of breastfeeding
Exogenous hormone consumption	Oral contraceptive medication, menopausal hormone therapy, and hormone replacement therapy
Nutrition	Alcohol consumption, high trans-fat content food consumption, and smoking habit
Anthropometry	Obesity, high body mass index (BMI), high weight gain, and body fat distribution
Physical inactivity	Lack of routine exercise or bodywork
History of breast pathologies	Atypical hyperplasia, lobular carcinoma in situ, and high mammographic density
Exposure to therapeutic radiation	Therapeutic chest radiation for the treatment of Hodgkin’s disease

**Table 2:** Summary of the latest approved viral gene therapy [12,21].

Name	Type of Therapy/Vector	Function	Approval from	Year of Approval
GlyberaR	Adeno-associated virus based	Familial lipoprotein lipase deficiency	European Medicines Agency (EMA)	2012
IMLYGICR	Genetically modified herpes simplex virus type 1	Local treatment of unresectable lesions in patients with melanoma,	US Food and Drug Administration (FDA)	2015
StrimvelisR	γ-retrovirus-based therapy	Treatment of severe combined immunodeficiency due to adenosine deaminase deficiency (ADA-SCID)	European Medicines Agency (EMA)	2016
KYMRIAHR	CD19-directed genetically modified autologous CAR T cell immunotherapies	1—Treatment of non-Hodgkin lymphoma 2—Treatment of acute lymphoblastic leukaemia.	US Food and Drug Administration (FDA)	2017
YESCARTAR	CD19-directed genetically modified autologous CAR T cell immunotherapies	Treatment of non-Hodgkin lymphoma	US Food and Drug Administration (FDA)	2017
LUXTURNAR	AAV-based gene therapy	Treatment of biallelic *RPE65* mutation-associated retinal dystrophy.	US Food and Drug Administration (FDA)	2017

**Table 3:** The approved liposome-based delivery systems are used as treatments against breast cancer [47].

Use of Liposomes in Breast Cancer Delivery System [48,49]				
Name	Year Approved	Content within	Applications	Lipid Composition
Doxil	1995	Doxorubicin	Ovarian, breast cancer, and Kaposi’s sarcoma	HSPC:Chol:PEG 2000-DSPE
Myocet	2000	Doxorubicin	Metastatic breast cancer	EPC:Chol
Lipo-dox	2012	Doxorubicin	Breast and ovarian cancer	DSPC:Chol:PEG 2000-DSPE

HSPC = L-α-phosphatidylcholine, hydrogenated (Soy), Chol = Cholesterol, PEG 2000-DSPE = PEGylated derivative of 1,2-distearoyl-sn-glycero-3-PE, DSPC = Distearoylphosphatidylcholine, EPC = 1,2-dioleoyl-sn-glycero-3-ethylphosphocholine.

**Table 4:** Clinical studies of liposome-based breast cancer gene therapy (data obtained from www.clinicaltrials.gov (accessed on 7 July 2022)).

Title of Clinical Trial	Status	Phase of Study	Strategy	Target Patients	Investigators	References
Phase II, Single Arm, Single Institution Clinical Trial of Docetaxel and Doxorubicin in Combination with Local Administration of INGN 201 (Ad5CMV-p53) in Locally Advanced Breast Cancer	Completed, 2004	Phase II	Combining liposomal chemotherapy drugs (docetaxel and doxorubicin hydrochloride) with gene (Ad5CMV-p53) in treating patients who have stage III or stage IV breast cancer	18 Years and older (male/female) Stage III and IV breast cancer patient	Jill Van Warthood (Introgen Therapeutics) United States, Texas	[52]
A Pilot Study of SGT-53 With Carboplatin and Pembrolizumab in Metastatic Triple Negative Inflammatory Breast Cancer	Starting on 30 October 2021	Phase I	Transferrin Receptor-Targeted Liposomal p53 cDNA, pembrolizumab, and carboplatin may help control the disease in patients with triple-negative inflammatory breast cancer.	18 Years and older (female) inflammatory breast cancer patient	Massimo Cristofanilli, FACP (Northwestern University) United States, Illinois	[53]

**Table 5:** Types of common cationic lipids used in formulating cationic liposomes.

Structure	Abbreviation	Name
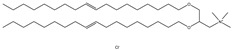	DOTMA	1,2-di-O-octadecenyl-3-trimethylammonium propane
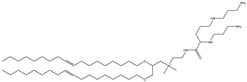	DOSPA (derived from DOTMA)	(+)-N,N-dimethyl-N-[2-(spermine car-boxamido) ethyl]-2,3-bis(dioleyloxy)-1-propaniminium pentahy- drochloride
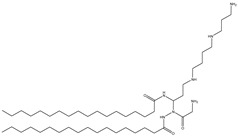	DOGS (derived from DOSPA)	Dioctadecylamidoglycyl spermine
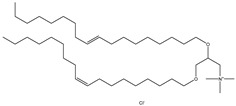	DOTAP	N-[1-(2,3-Dioleoyloxy) propyl]-N, N, N-trimethylammonium chloride
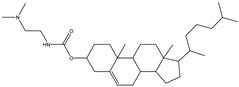	DC-Chol	3ß-[N-(N′, N′-dimethylaminoethane)-carbamoyl] cholesterol hydrochloride
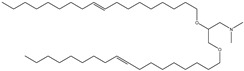	DODMA	1,2-dioleyloxy-3-dimethylaminopropane
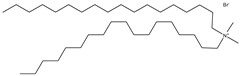	DDAB	Dimethyldioctadecylammonium (Bromide Salt)
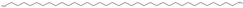	Octadecylamine	Stearylamine, 1-Aminooctadecane

**Table 6:** Types of common anionic lipids used in formulating anionic liposomes.

Structure	Abbreviation	Name
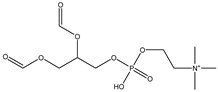	PC	Phosphatidyl-choline
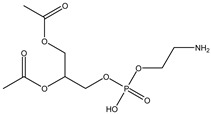	PE	Phosphatidyl-ethanolamine
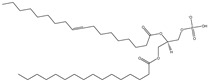	PA	L-α-phosphatidic acid (Egg, Chicken) (sodium salt)
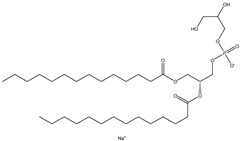	PG	1,2-dimyristoyl-sn-glycero-3-phospho-(1′-rac-glycerol) (sodium salt)
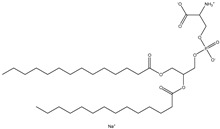	PS	1,2-dimyristoyl-sn-glycero-3-phospho-L-serine (sodium salt)

**Table 7:** Types of common neutral lipids used in formulating neutral liposomes for drug and gene delivery.

Structure	Abbreviation	Name
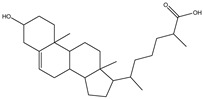	Cholesterol	3β-Hydroxy-5-cholestene, 5-Cholesten-3β-ol, Cholesterol
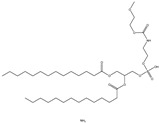	DOPE	1,2-dioleoyl-*sn*-glycero-3-phosphoethanolamine
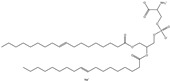	EPC (Cl salt)	1,2-dioleoyl-*sn*-glycero-3-ethylphosphocholine (chloride salt)
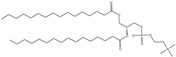	DPPC	1,2-dipalmitoyl-*sn*-glycero-3-phosphocholine
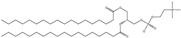	DSPC	1,2-distearoyl-*sn*-glycero-3-phosphocholine
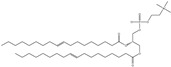	DOPC	1,2-dioleoyl-*sn*-glycero-3-phosphocholine

**Table 8:** Successful liposome formulations tested on breast cancer cell lines with gene cargo and their type of study reported in publications.

Lipid Nanomaterials	Type of Liposome	Type of Study	Cargo	Outcome	Cell Line	Year	Reference
DOTAP and cholesterol	Cationic	In Vitro	Paclitaxel and Protein-coding gene siRNA (si-Plk1)	Combined treatment that eliminates breast cancer cells better by 35–60%.	MCF-7 and MDA-MB-231	2018	[82]
DOPE, DOTAP, PC, and cholesterol	Cationic	In Vitro and In Vivo	Docetaxel and silencing gene shRNA (Sirtuin 1)	More efficient in inducing cancer cell apoptosis and size tumour reduced	MCF-7 and MDA-MB-231	2021	[83]
DOPE, DOTAP and well-defined synthetic multifunctional peptide, DEN-K(GALA)-TAT-K(STR)-CTP	Cationic	In Vitro	siRNA B-cell lymphoma 2 (BCL2)	Efficient cell internalisation and higher levels of gene expression	MCF-7	2017	[84]
L-α-phosphatidylcholine (PC), palmitoyl-snGlycero-3-Phopshoethanolamine (DPPE) and cholesterol (CHOL) with HA	Neutral	In Vitro	microRNA tumour suppressor (miR-125a-5p)	Significantly inhibited HER2 expression as well as cell proliferation and migration in the 21MT-1 cell line	21MT-1	2016	[116]
DOPG/DOPE and calcium ion	Anionic	In Vitro	siRNA (anti-eGFP siRNA)	Maximum silencing, low cytotoxic, stable and high efficiency in serum, efficient intracellular uptake and endosomal escape.	MDA-MB-231	2012	[58]
Poly (ethylene glycol)-block-poly (D, L-lactide) (PEG5K-PLA11K) and the cationic lipid N, N-bis(2-hydroxyethyl)-N-methyl-N-(2- cholesteryoxycarbonyl-aminoethyl) ammonium bromide (BHEM-Chol)	Cationic	In Vitro and In Vivo	siRNA, cyclin-dependent kinase 1 (CDK1)	Synthetic lethality in TNBC cells with high cMyc expression in mouse xenograft	SUM149	2014	[119]
Anti-CD44 aptamer conjugate (Liposomes) DPPC, cholesterol, and DSPE-PEG	Cationic	In Vitro and In Vivo	siRNA Firefly luciferase with protamine	Functionalised with anti-CD44 aptamer in the TNBC model exhibits gene silencing.	(MDAMB-231)	2017	[117]
DOPE, cholesterol and DMPG liposome conjugated with Fab’ antibody against heparin-binding EGF-like growth factor	Anionic	In Vitro and In Vivo	siRNA, polo-like kinase 1 (PLK 1)	Suppression of polo-like kinase 1 expression; tumour growth reduction	(MDA-MB-231)	2018	[118]

**Table 9:** Summary of the advantages and disadvantages of cationic, anionic and neutral liposomes used as a non-viral gene carrier, specifically for gene therapy.

Types of Liposomes	Advantages	Disadvantages
Cationic	Naturally occurring electrostatic interaction with negatively charged nucleic acid Cell binding ability with anionic endosomal membranes Better transfection efficiency Stable when administered into the bloodstream Better cellular uptake	High toxicity Low serum stability Low circulation time
Anionic	Low cytotoxicity Stable in the presence of serum Better transfection efficiency	Low cellular uptake Low nucleic acid encapsulation efficiency Less stable when administered into the bloodstream Low circulation time
Neutral	Stable when administered into the bloodstream Long circulation time Low cytotoxicity	Low cellular uptake Low nucleic acid encapsulation efficiency Low serum stability Low transfection ability

## Data Availability

Not applicable.

## References

[1] Ferlay J., Colombet M., Soerjomataram I., Parkin D.M., Piñeros M., Znaor A., Bray F. (2021). Cancer Statistics for the Year 2020: An Overview. Int. J. Cancer.

[2] Ferlay J., Shin H.R., Bray F., Forman D., Mathers C., Parkin D.M. (2010). Estimates of Worldwide Burden of Cancer in 2008: GLOBOCAN 2008. Int. J. Cancer.

[3] Sung H., Ferlay J., Siegel R.L., Laversanne M., Soerjomataram I., Jemal A., Bray F. (2021). Global Cancer Statistics 2020: GLOBOCAN Estimates of Incidence and Mortality Worldwide for 36 Cancers in 185 Countries. CA Cancer J. Clin..

[4] Bray F., Ferlay J., Soerjomataram I., Siegel R.L., Torre L.A., Jemal A. (2018). Global Cancer Statistics 2018: GLOBOCAN Estimates of Incidence and Mortality Worldwide for 36 Cancers in 185 Countries. CA Cancer J. Clin..

[5] Jemal A., Bray F., Center M.M., Ferlay J., Ward E., Forman D. (2011). Global Cancer Statistics. CA Cancer J. Clin..

[6] Britt K.L., Cuzick J., Phillips K.A. (2020). Key Steps for Effective Breast Cancer Prevention. Nat. Rev. Cancer.

[7] Waks A.G., Winer E.P. (2019). Breast Cancer Treatment: A Review. J. Am. Med. Assoc..

[8] Tong C.W.S., Wu M., Cho W.C.S., To K.K.W. (2018). Recent Advances in the Treatment of Breast Cancer. Front. Oncol..

[9] Fraguas-Sánchez A.I., Martín-Sabroso C., Fernández-Carballido A., Torres-Suárez A.I. (2019). Current Status of Nanomedicine in the Chemotherapy of Breast Cancer. Cancer Chemother. Pharmacol..

[10] Toy W., Shen Y., Won H., Green B., Sakr R.A., Will M., Li Z., Gala K., Fanning S., King T.A. (2013). ESR1 Ligand-Binding Domain Mutations in Hormone-Resistant Breast Cancer. Nat. Genet..

[11] Greenlee H., Kwan M.L., Ergas I.J., Sherman K.J., Krathwohl S.E., Bonnell C., Lee M.M., Kushi L.H. (2009). Complementary and Alternative Therapy Use before and after Breast Cancer Diagnosis: The Pathways Study. Breast Cancer Res. Treat..

[12] Anguela X.M., High K.A. (2019). Entering the Modern Era of Gene Therapy. Annu. Rev. Med..

[13] Komor A.C., Badran A.H., Liu D.R. (2017). CRISPR-Based Technologies for the Manipulation of Eukaryotic Genomes. Cell.

[14] Moghaddam B., Ali M.H., Wilkhu J., Kirby D.J., Mohammed A.R., Zheng Q., Perrie Y. (2011). The Application of Monolayer Studies in the Understanding of Liposomal Formulations. Int. J. Pharm..

[15] Keles E., Song Y., Du D., Dong W.J., Lin Y. (2016). Recent Progress in Nanomaterials for Gene Delivery Applications. Biomater. Sci..

[16] Cavazzana-Calvo M., Hacein-Bey S., de Saint Basile G., Gross F., Yvon E., Nusbaum P., Selz F., Hue C., Certain S., Casanova J.L. (2000). Gene Therapy of Human Severe Combined Immunodeficiency (SCID)-X1 Disease. Science.

[17] Hollon T. (2000). Researchers and Regulators Reflect on First Gene Therapy Death. Nat. Med..

[18] Aiuti A., Cattaneo F., Galimberti S., Benninghoff U., Cassani B., Callegaro L., Scaramuzza S., Andolfi G., Mirolo M., Brigida I. (2009). Gene Therapy for Immunodeficiency Due to Adenosine Deaminase Deficiency. N. Engl. J. Med..

[19] Notarangelo L., Casanova J.L., Fischer A., Puck J., Rosen F., Seger R., Geha R. (2004). Primary Immunodeficiency Diseases: An Update. J. Allergy Clin. Immunol..

[20] Patil S., Gao Y.G., Lin X., Li Y., Dang K., Tian Y., Zhang W.J., Jiang S.F., Qadir A., Qian A.R. (2019). The Development of Functional Non-Viral Vectors for Gene Delivery. Int. J. Mol. Sci..

[21] Belete T.M. (2021). The Current Status of Gene Therapy for the Treatment of Cancer. Biologics.

[22] Khan I., Saeed K., Khan I. (2019). Nanoparticles: Properties, Applications and Toxicities. Arab. J. Chem..

[23] Srivastava A., Mallela K.M.G., Deorkar N., Brophy G. (2021). Manufacturing Challenges and Rational Formulation Development for AAV Viral Vectors. J. Pharm. Sci..

[24] Bangham A.D., Hill M.W., Miller N.G.A. (1974). Preparation and Use of Liposomes as Models of Biological Membranes. Methods in Membrane Biology.

[25] Balazs D.A., Godbey W.T. (2011). Liposomes for Use in Gene Delivery. J. Drug Deliv..

[26] Gregoriadis G. (2016). Liposomes in Drug Delivery: How It All Happened. Pharmaceutics.

[27] Tavares Luiz M., Aparecida J., Dutra P., Bueno Tofani L., Cavalcante De Araújo J.T., Delello L., Filippo D., Marchetti J.M., Chorilli M. (2022). Targeted Liposomes: A Nonviral Gene Delivery System for Cancer Therapy. Pharmaceutics.

[28] Schepelmann S., Springer C.J. (2008). Gene Therapy for Cancer. Br. J. Cancer.

[29] Yang B., Song B.P., Shankar S., Guller A., Deng W. (2021). Recent Advances in Liposome Formulations for Breast Cancer Therapeutics. Cell. Mol. Life Sci..

[30] Dastjerd N.T., Valibeik A., Rahimi Monfared S., Goodarzi G., Moradi Sarabi M., Hajabdollahi F., Maniati M., Amri J., Samavarchi Tehrani S. (2022). Gene Therapy: A Promising Approach for Breast Cancer Treatment. Cell Biochem. Funct..

[31] Liu C., Zhang L., Zhu W., Guo R., Sun H., Chen X., Deng N. (2020). Barriers and Strategies of Cationic Liposomes for Cancer Gene Therapy. Mol. Ther. Methods Clin. Dev..

[32] Chong Z.X., Yeap S.K., Ho W.Y. (2021). Transfection Types, Methods and Strategies: A Technical Review. PeerJ.

[33] Kim T.K., Eberwine J.H. (2010). Mammalian Cell Transfection: The Present and the Future. Anal. Bioanal. Chem..

[34] Ding S., O’Banion C.P., Welfare J.G., Lawrence D.S. (2018). Cellular Cyborgs: On the Precipice of a Drug Delivery Revolution. Cell Chem. Biol..

[35] Zhi D., Bai Y., Yang J., Cui S., Zhao Y., Chen H., Zhang S. (2018). A Review on Cationic Lipids with Different Linkers for Gene Delivery. Adv. Colloid Interface Sci..

[36] Frolov V.A., Shnyrova A.v., Zimmerberg J. (2011). Lipid Polymorphisms and Membrane Shape. Cold Spring Harb. Perspect. Biol..

[37] Hafez I.M., Cullis P.R. (2001). Roles of Lipid Polymorphism in Intracellular Delivery. Adv. Drug Deliv. Rev..

[38] Moosavian S.A., Sahebkar A. (2019). Aptamer-Functionalized Liposomes for Targeted Cancer Therapy. Cancer Lett..

[39] Key J., Palange A.L., Gentile F., Aryal S., Stigliano C., di Mascolo D., de Rosa E., Cho M., Lee Y., Singh J. (2015). Soft Discoidal Polymeric Nanoconstructs Resist Macrophage Uptake and Enhance Vascular Targeting in Tumors. ACS Nano.

[40] Anselmo A.C., Zhang M., Kumar S., Vogus D.R., Menegatti S., Helgeson M.E., Mitragotri S. (2015). Elasticity of Nanoparticles Influences Their Blood Circulation, Phagocytosis, Endocytosis, and Targeting. ACS Nano.

[41] Hosta-Rigau L., Shimoni O., Städler B., Caruso F. (2013). Hydrogels: Advanced Subcompartmentalized Microreactors: Polymer Hydrogel Carriers Encapsulating Polymer Capsules and Liposomes (Small 21/2013). Small.

[42] Beltrán-Gracia E., López-Camacho A., Higuera-Ciapara I., Velázquez-Fernández J.B., Vallejo-Cardona A.A. (2019). Nanomedicine Review: Clinical Developments in Liposomal Applications. Cancer Nanotechnol..

[43] Nkanga C.I., Krause R.W.M. (2019). Encapsulation of Isoniazid-Conjugated Phthalocyanine-In-Cyclodextrin-In-Liposomes Using Heating Method. Sci. Rep..

[44] Shukla S., Haldorai Y., Hwang S.K., Bajpai V.K., Huh Y.S., Han Y.K. (2017). Current Demands for Food-Approved Liposome Nanoparticles in Food and Safety Sector. Front. Microbiol..

[45] Filipczak N., Pan J., Yalamarty S.S.K., Torchilin V.P. (2020). Recent Advancements in Liposome Technology. Adv. Drug Deliv. Rev..

[46] Rafiyath S.M., Rasul M., Lee B., Wei G., Lamba G., Liu D. (2012). Comparison of Safety and Toxicity of Liposomal Doxorubicin vs. Conventional Anthracyclines: A Meta-Analysis. Exp. Hematol. Oncol..

[47] Kim E.-M., Jeong H.-J. (2021). Liposomes: Biomedical Applications. Chonnam Med. J..

[48] Bobo D., Robinson K.J., Islam J., Thurecht K.J., Corrie S.R. (2016). Nanoparticle-Based Medicines: A Review of FDA-Approved Materials and Clinical Trials to Date. Pharm. Res..

[49] Anselmo A.C., Mitragotri S. (2019). Nanoparticles in the Clinic: An Update. Bioeng. Transl. Med..

[50] Patil S.D., Rhodes D.G., Burgess D.J. (2004). Anionic Liposomal Delivery System for DNA Transfection. AAPS J..

[51] Giulimondi F., Digiacomo L., Pozzi D., Palchetti S., Vulpis E., Capriotti A.L., Chiozzi R.Z., Laganà A., Amenitsch H., Masuelli L. (2019). Interplay of Protein Corona and Immune Cells Controls Blood Residency of Liposomes. Nat. Commun..

[52] Chemotherapy Combined with Gene Therapy in Treating Patients Who Have Stage III or Stage IV Breast Cancer—Full Text View—ClinicalTrials.gov. https://www.clinicaltrials.gov/ct2/show/NCT00044993?term=liposome+gene+therapy&cond=breast+cancer&draw=2&rank=1.

[53] SGT-53, Carboplatin, and Pembrolizumab for the Treatment of Metastatic Triple Negative Inflammatory Breast Cancer—Full Text View—ClinicalTrials.gov. https://www.clinicaltrials.gov/ct2/show/NCT05093387?term=liposome+gene+therapy&cond=breast+cancer&draw=2&rank=2.

[54] Wonder E., Simón-Gracia L., Scodeller P., Majzoub R.N., Kotamraju V.R., Ewert K.K., Teesalu T., Safinya C.R. (2018). Competition of Charge-Mediated and Specific Binding by Peptide-Tagged Cationic Liposome-DNA Nanoparticles in Vitro and in Vivo. Biomaterials.

[55] Simões S., Filipe A., Faneca H., Mano M., Penacho N., Düzgünes N., de Lima M.P. (2005). Cationic Liposomes for Gene Delivery. Expert Opin. Drug Deliv..

[56] Kang J.H., Jang W.Y., Ko Y.T. (2017). The Effect of Surface Charges on the Cellular Uptake of Liposomes Investigated by Live Cell Imaging. Pharm. Res..

[57] Francia V., Yang K., Deville S., Reker-Smit C., Nelissen I., Salvati A. (2019). Corona Composition Can Affect the Mechanisms Cells Use to Internalize Nanoparticles. ACS Nano.

[58] Kapoor M., Burgess D.J. (2012). Efficient and Safe Delivery of SiRNA Using Anionic Lipids: Formulation Optimization Studies. Int. J. Pharm..

[59] Pisani M., Mobbili G., Bruni P. (2011). Neutral Liposomes and DNA Transfection. Non-Viral Gene Therapy.

[60] Hattori Y., Hara E., Shingu Y., Minamiguchi D., Nakamura A., Arai S., Ohno H., Kawano K., Fujii N., Yonemochi E. (2015). SiRNA Delivery into Tumor Cells by Cationic Cholesterol Derivative-Based Nanoparticles and Liposomes. Biol. Pharm. Bull..

[61] Inglut C.T., Sorrin A.J., Kuruppu T., Vig S., Cicalo J., Ahmad H., Huang H.C. (2020). Immunological and Toxicological Considerations for the Design of Liposomes. Nanomaterials.

[62] Cui S., Wang Y., Gong Y., Lin X., Zhao Y., Zhi D., Zhou Q., Zhang S. (2018). Correlation of the Cytotoxic Effects of Cationic Lipids with Their Headgroups. Toxicol. Res..

[63] Zhi D., Zhang S., Wang B., Zhao Y., Yang B., Yu S. (2010). Transfection Efficiency of Cationic Lipids with Different Hydrophobic Domains in Gene Delivery. Bioconjug. Chem..

[64] Zhu Y., Meng Y., Zhao Y., Zhu J., Xu H., Zhang E., Shi L., Du L., Liu G., Zhang C. (2019). Toxicological Exploration of Peptide-Based Cationic Liposomes in SiRNA Delivery. Colloids Surf. B Biointerfaces.

[65] Vermeulen L.M.P., de Smedt S.C., Remaut K., Braeckmans K. (2018). The Proton Sponge Hypothesis: Fable or Fact?. Eur. J. Pharm. Biopharm..

[66] Li M., Du C., Guo N., Teng Y., Meng X., Sun H., Li S., Yu P., Galons H. (2019). Composition Design and Medical Application of Liposomes. Eur. J. Med. Chem..

[67] Haghiralsadat F., Amoabediny G., Naderinezhad S., Forouzanfar T., Helder M.N., Zandieh-Doulabi B. (2018). Preparation of PEGylated Cationic Nanoliposome-SiRNA Complexes for Cancer Therapy. Artif. Cells Nanomed. Biotechnol..

[68] Jiang Q., Yue D., Nie Y., Xu X., He Y., Zhang S., Wagner E., Gu Z. (2016). Specially-Made Lipid-Based Assemblies for Improving Transmembrane Gene Delivery: Comparison of Basic Amino Acid Residue Rich Periphery. Mol. Pharm..

[69] Wang J., Ye X., Ni H., Zhang J., Ju S., Ding W. (2019). Transfection Efficiency Evaluation and Endocytosis Exploration of Different Polymer Condensed Agents. DNA Cell. Biol..

[70] Rezaee M., Oskuee R.K., Nassirli H., Malaekeh-Nikouei B. (2016). Progress in the Development of Lipopolyplexes as Efficient Non-Viral Gene Delivery Systems. J. Control. Release.

[71] Behzadi S., Serpooshan V., Tao W., Hamaly M.A., Alkawareek M.Y., Dreaden E.C., Brown D., Alkilany A.M., Farokhzad O.C., Mahmoudi M. (2017). Cellular Uptake of Nanoparticles: Journey inside the Cell. Chem. Soc. Rev..

[72] Lin J., Alexander-Katz A. (2013). Cell Membranes Open “Doors” for Cationic Nanoparticles/ Biomolecules: Insights into Uptake Kinetics. ACS Nano.

[73] Zhang L., Yang X., Lv Y., Xin X., Qin C., Han X., Yang L., He W., Yin L. (2017). Cytosolic Co-Delivery of MiRNA-34a and Docetaxel with Core-Shell Nanocarriers via Caveolae-Mediated Pathway for the Treatment of Metastatic Breast Cancer. Sci. Rep..

[74] Kumar V., Singh R., Chaudhuri A. (2003). Cationic Transfection Lipids in Gene Therapy: Successes, Set-Backs, Challenges and Promises. Curr. Med. Chem..

[75] Li T., He J., Horvath G., Próchnicki T., Latz E., Takeoka S. (2018). Lysine-Containing Cationic Liposomes Activate the NLRP3 Inflammasome: Effect of a Spacer between the Head Group and the Hydrophobic Moieties of the Lipids. Nanomedicine.

[76] Tomori Y., Iijima N., Hinuma S., Ishii H., Takumi K., Takai S., Ozawa H. (2018). Morphological Analysis of Trafficking and Processing of Anionic and Cationic Liposomes in Cultured Cells. Acta Histochem. Cytochem..

[77] Audouy S., Hoekstra D. (2001). Cationic Lipid-Mediated Transfection in Vitro and in Vivo (Review). Mol. Membr. Biol..

[78] Dokka S., Toledo D., Shi X., Castranova V., Rojanasakul Y. (2000). Oxygen Radical-Mediated Pulmonary Toxicity Induced by Some Cationic Liposomes. Pharm. Res..

[79] Neves L.F., Duan J., Voelker A., Khanal A., McNally L., Steinbach-Rankins J., Ceresa B.P. (2016). Preparation and Optimization of Anionic Liposomes for Delivery of Small Peptides and CDNA to Human Corneal Epithelial Cells. J. Microencapsul..

[80] Hosseini E.S., Nikkhah M., Hosseinkhani S. (2019). Cholesterol-Rich Lipid-Mediated Nanoparticles Boost of Transfection Efficiency, Utilized for Gene Editing by CRISPR-Cas9. Int. J. Nanomed..

[81] Barenholz Y. (2012). Doxil®—The First FDA-Approved Nano-Drug: Lessons Learned. J. Control. Release.

[82] Bulbake U., Kommineni N., Bryszewska M., Ionov M., Khan W. (2018). Cationic Liposomes for Co-Delivery of Paclitaxel and Anti-Plk1 SiRNA to Achieve Enhanced Efficacy in Breast Cancer. J. Drug Deliv. Sci. Technol..

[83] Swami R., Kumar Y., Chaudhari D., Katiyar S.S., Kuche K., Katare P.B., Banerjee S.K., Jain S. (2021). PH Sensitive Liposomes Assisted Specific and Improved Breast Cancer Therapy Using Co-Delivery of SIRT1 ShRNA and Docetaxel. Mater. Sci. Eng. C.

[84] Wan Y., Dai W., Nevagi R.J., Toth I., Moyle P.M. (2017). Multifunctional Peptide-Lipid Nanocomplexes for Efficient Targeted Delivery of DNA and SiRNA into Breast Cancer Cells. Acta Biomater..

[85] Akhtar S., Basu S., Wickstrom E., Juliano R.L. (1991). Interactions of Antisense DNA Oligonucleotide Analogs with Phospholipid Membranes (Liposomes). Nucleic Acids Res..

[86] Lakkaraju A., Dubinsky J.M., Low W.C., Rahman Y.E. (2001). Neurons Are Protected from Excitotoxic Death by P53 Antisense Oligonucleotides Delivered in Anionic Liposomes. J. Biol. Chem..

[87] Forssen E.A., Tökès Z.A. (1981). Use of Anionic Liposomes for the Reduction of Chronic Doxorubicin-Induced Cardiotoxicity. Proc. Natl. Acad. Sci. USA.

[88] Wang L.-L., Feng C.-L., Zheng W.-S., Huang S., Zhang W.-X., Wu H.-N., Zhan Y., Han Y.-X., Wu S., Jiang J.-D. (2017). Tumor-Selective Lipopolyplex Encapsulated Small Active RNA Hampers Colorectal Cancer Growth in Vitro and in Orthotopic Murine. Biomaterials.

[89] Chen Z., Place R.F., Jia Z.J., Pookot D., Dahiya R., Li L.C. (2008). Antitumor Effect of DsRNA-Induced P21(WAF1/CIP1) Gene Activation in Human Bladder Cancer Cells. Mol. Cancer Ther..

[90] Bozzuto G., Molinari A. (2015). Liposomes as Nanomedical Devices. Int J Nanomed..

[91] Lungwitz U., Breunig M., Blunk T., Göpferich A. (2005). Polyethylenimine-Based Non-Viral Gene Delivery Systems. Eur. J. Pharm. Biopharm..

[92] Ewe A., Schaper A., Barnert S., Schubert R., Temme A., Bakowsky U., Aigner A. (2014). Storage Stability of Optimal Liposome-Polyethylenimine Complexes (Lipopolyplexes) for DNA or SiRNA Delivery. Acta Biomater..

[93] Jerzykiewicz J., Czogalla A. (2021). Polyethyleneimine-Based Lipopolyplexes as Carriers in Anticancer Gene Therapies. Materials.

[94] Kurosaki T., Kawakami S., Higuchi Y., Suzuki R., Maruyama K., Sasaki H., Yamashita F., Hashida M. (2014). Development of Anionic Bubble Lipopolyplexes for Efficient and Safe Gene Transfection with Ultrasound Exposure in Mice. J. Control. Release.

[95] Chen M., Zeng Z., Qu X., Tang Y., Long Q., Feng X. (2015). Biocompatible Anionic Polyelectrolyte for Improved Liposome Based Gene Transfection. Int. J. Pharm..

[96] Nchinda G., Überla K., Zschörnig O. (2002). Characterization of Cationic Lipid DNA Transfection Complexes Differing in Susceptability to Serum Inhibition. BMC Biotechnol..

[97] van Tran V., Moon J.Y., Lee Y.C. (2019). Liposomes for Delivery of Antioxidants in Cosmeceuticals: Challenges and Development Strategies. J. Control. Release.

[98] Sawant R.R., Torchilin V.P. (2012). Challenges in Development of Targeted Liposomal Therapeutics. AAPS J..

[99] Bajoria R., Fisk N.M., Contractor S.F. (1997). Liposomal Thyroxine: A Noninvasive Model for Transplacental Fetal Therapy. J. Clin. Endocrinol. Metab..

[100] Figueiredo S., Cabral R., Luís D., Fernandes A.R., Baptista P.V. (2014). Conjugation of Gold Nanoparticles and Liposomes for Combined Vehicles of Drug Delivery in Cancer. Nanomedicine.

[101] Ibaraki H., Takeda A., Arima N., Hatakeyama N., Takashima Y., Seta Y., Kanazawa T. (2021). In Vivo Fluorescence Imaging of Passive Inflammation Site Accumulation of Liposomes via Intravenous Administration Focused on Their Surface Charge and Peg Modification. Pharmaceutics.

[102] Nie Y., Ji L., Ding H., Xie L., Li L., He B., Wu Y., Gu Z. (2012). Cholesterol Derivatives Based Charged Liposomes for Doxorubicin Delivery: Preparation, in Vitro and in Vivo Characterization. Theranostics.

[103] Lasic D.D. (2019). Liposomes in Gene Delivery.

[104] Srinivasan C., Burgess D.J. (2009). Optimization and Characterization of Anionic Lipoplexes for Gene Delivery. J. Control. Release.

[105] Lee R.J., Huang L. (1996). Folate-Targeted, Anionic Liposome-Entrapped Polylysine-Condensed DNA for Tumor Cell-Specific Gene Transfer. J. Biol. Chem..

[106] Krasnici S., Werner A., Eichhorn M.E., Schmitt-Sody M., Pahernik S.A., Sauer B., Schulze B., Teifel M., Michaelis U., Naujoks K. (2003). Effect of the Surface Charge of Liposomes on Their Uptake by Angiogenic Tumor Vessels. Int. J. Cancer.

[107] Digiacomo L., Pozzi D., Palchetti S., Zingoni A., Caracciolo G. (2020). Impact of the Protein Corona on Nanomaterial Immune Response and Targeting Ability. Wiley Interdiscip. Rev. Nanomed. Nanobiotechnol..

[108] Channarong S., Chaicumpa W., Sinchaipanid N., Mitrevej A. (2011). Development and Evaluation of Chitosan-Coated Liposomes for Oral DNA Vaccine: The Improvement of Peyer’s Patch Targeting Using a Polyplex-Loaded Liposomes. AAPS PharmSciTech.

[109] Chang K., Chang F.H., Chen M.H. (2019). Developing a Novel Cholesterol-Based Nanocarrier with High Transfection Efficiency and Serum Compatibility for Gene Therapy. J. Formos. Med. Assoc..

[110] Nobs L., Buchegger F., Gurny R., Allémann E. (2004). Current Methods for Attaching Targeting Ligands to Liposomes and Nanoparticles. J. Pharm. Sci..

[111] Hillaireau H., Couvreur P. (2009). Nanocarriers’ Entry into the Cell: Relevance to Drug Delivery. Cell. Mol. Life Sci..

[112] Montizaan D., Yang K., Reker-Smit C., Salvati A. (2020). Comparison of the Uptake Mechanisms of Zwitterionic and Negatively Charged Liposomes by HeLa Cells. Nanomedicine.

[113] Bruni P., Pisani M., Amici A., Marchini C., Montani M., Francescangeli O. (2006). Self-Assembled Ternary Complexes of Neutral Liposomes, Deoxyribonucleic Acid, and Bivalent Metal Cations. Promising Vectors for Gene Transfer?. Appl. Phys. Lett..

[114] Caracciolo G., Pozzi D., Caminiti R., Marchini C., Montani M., Amici A., Amenitsch H. (2007). Transfection Efficiency Boost by Designer Multicomponent Lipoplexes. Biochim. Biophys. Acta (BBA)-Biomembr..

[115] Lange Y., Ye J., Steck T.L. (2014). Essentially All Excess Fibroblast Cholesterol Moves from Plasma Membranes to Intracellular Compartments. PLoS ONE.

[116] Hayward S.L., Francis D.M., Kholmatov P., Kidambi S. (2016). Targeted Delivery of MicroRNA125a-5p by Engineered Lipid Nanoparticles for the Treatment of HER2 Positive Metastatic Breast Cancer. J. Biomed. Nanotechnol..

[117] Alshaer W., Hillaireau H., Vergnaud J., Mura S., Deloménie C., Sauvage F., Ismail S., Fattal E. (2018). Aptamer-Guided SiRNA-Loaded Nanomedicines for Systemic Gene Silencing in CD-44 Expressing Murine Triple-Negative Breast Cancer Model. J. Control. Release.

[118] Okamoto A., Asai T., Hirai Y., Shimizu K., Koide H., Minamino T., Oku N. (2018). Systemic Administration of SiRNA with Anti-HB-EGF Antibody-Modified Lipid Nanoparticles for the Treatment of Triple-Negative Breast Cancer. Mol. Pharm..

[119] Liu Y., Zhu Y.H., Mao C.Q., Dou S., Shen S., Tan Z.b., Wang J. (2014). Triple Negative Breast Cancer Therapy with CDK1 SiRNA Delivered by Cationic Lipid Assisted PEG-PLA Nanoparticles. J. Control. Release.

[120] Bulbake U., Doppalapudi S., Kommineni N., Khan W. (2017). Liposomal Formulations in Clinical Use: An Updated Review. Pharmaceutics.

